# Treatment Response in Kawasaki Disease Is Associated with Sialylation Levels of Endogenous but Not Therapeutic Intravenous Immunoglobulin G

**DOI:** 10.1371/journal.pone.0081448

**Published:** 2013-12-06

**Authors:** Shohei Ogata, Chisato Shimizu, Alessandra Franco, Ranim Touma, John T. Kanegaye, Biswa P. Choudhury, Natasha N. Naidu, Yutaka Kanda, Long T. Hoang, Martin L. Hibberd, Adriana H. Tremoulet, Ajit Varki, Jane C. Burns

**Affiliations:** 1 Department of Pediatrics, University of California San Diego, School of Medicine, La Jolla, California, United States of America; 2 Rady Children’s Hospital San Diego, San Diego, California, United States of America; 3 Department of Cellular and Molecular Medicine, Glycobiology Research and Training Center, University of San Diego, School of Medicine, La Jolla, California, United States of America; 4 Kyowa Hakko Kirin California, Inc., La Jolla, California, United States of America; 5 Division of Infectious Disease 1, Genome Institute of Singapore, Singapore, Singapore; 6 Division of Human Genetics, Genome Institute of Singapore, Singapore, Singapore; University of Leicester, United Kingdom

## Abstract

**Objectives:**

Although intravenous immunoglobulin (IVIG) is highly effective in Kawasaki disease (KD), mechanisms are not understood and 10-20% of patients are treatment-resistant, manifesting a higher rate of coronary artery aneurysms. Murine models suggest that α2-6-linked sialic acid (α2-6Sia) content of IVIG is critical for suppressing inflammation. However, pro-inflammatory states also up-regulate endogenous levels of β-galactoside:α2-6 sialyltransferase-I (ST6Gal-I), the enzyme that catalyzes addition of α2-6Sias to *N*-glycans. We asked whether IVIG failures correlated with levels of α2-6Sia on infused IVIG or on the patient’s own endogenous IgG.

**Methods:**

We quantified levels of α2-6Sia in infused IVIG and endogenous IgG from 10 IVIG-responsive and 10 resistant KD subjects using multiple approaches. Transcript levels of *ST6GAL1*, in patient whole blood and B cell lines were evaluated by RT-PCR. Plasma soluble (s)ST6Gal-I levels were measured by ELISA.

**Results:**

There was no consistent difference in median sialylation levels of infused IVIG between groups. However, α2-6Sia levels in endogenous IgG, *ST6GAL1* transcript levels, and ST6Gal-I protein in serum from IVIG-resistant KD subjects were lower than in responsive subjects at both pre-treatment and one-year time points (p <0.001, respectively).

**Conclusions:**

Our data indicate sialylation levels of therapeutic IVIG are unrelated to treatment response in KD. Rather, lower sialylation of endogenous IgG and lower blood levels of *ST6GALI* mRNA and ST6Gal-I enzyme predict therapy resistance. These differences were stable over time, suggesting a genetic basis. Because IVIG-resistance increases risk of coronary artery aneurysms, our findings have important implications for the identification and treatment of such individuals.

## Introduction

Kawasaki disease (KD) is an acute, self-limited vasculitis of unknown etiology that predominantly affects infants and children [1]. Coronary artery (CA) aneurysms, the most severe complication, occur in 25% of untreated children and may lead to ischemic heart disease, myocardial infarction, or sudden death [2]. While a single high dose of intravenous immunoglobulin (IVIG) terminates the fever and acute inflammation in most subjects and dramatically reduces the incidence of CA aneurysms,10 to 20% of KD patients are IVIG-resistant and have persistent or recrudescent fever at least 36 hours after the end of the initial IVIG infusion [3]. These subjects are at higher risk of developing CA abnormalities [4]. Neither the anti-inflammatory mechanism of IVIG nor the cause of IVIG-resistance is well understood.

**Figure 1:**
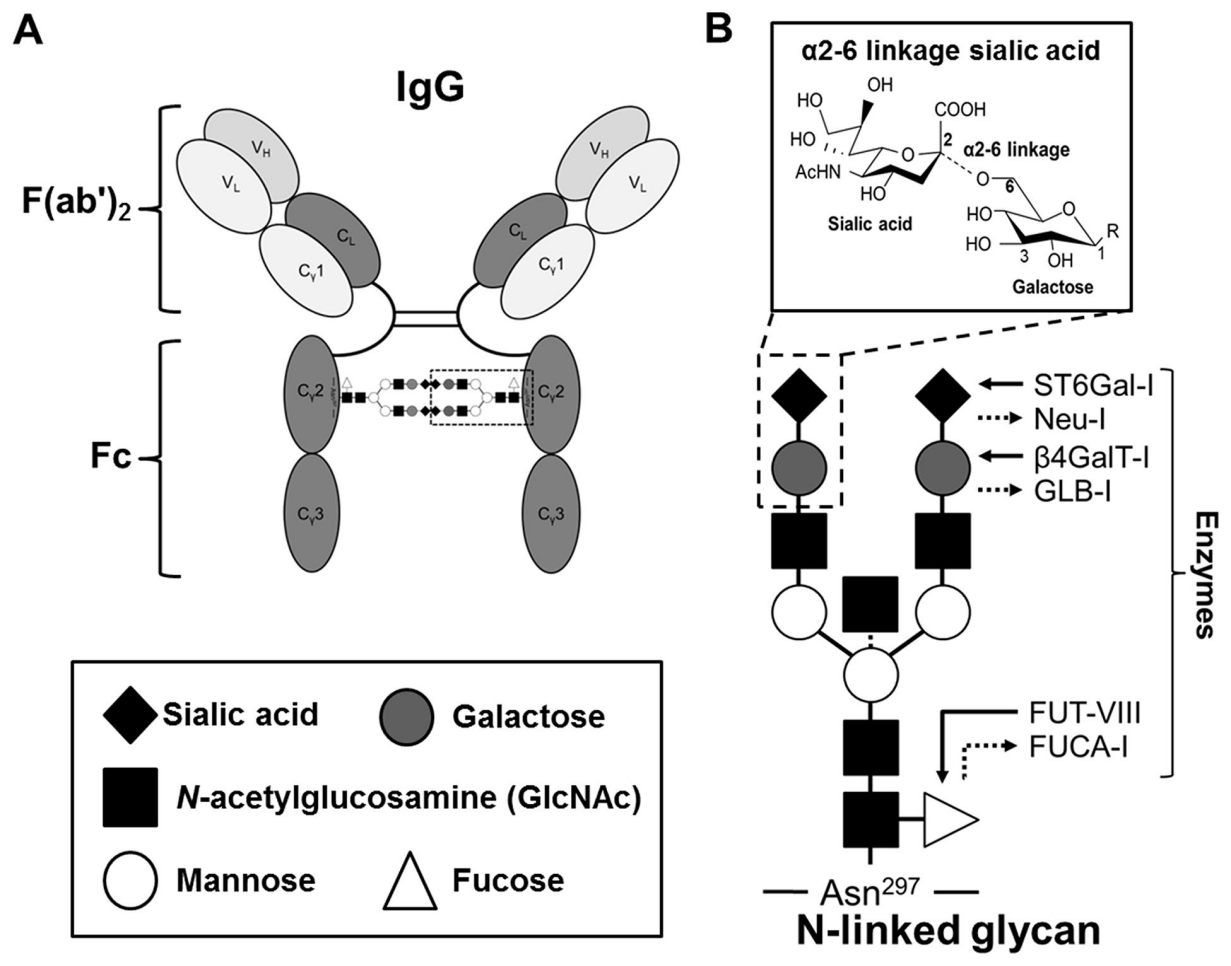
Structure of *N*-glycans in human IgG. (**A**) *N*-glycan with attached monosaccharaides on IgG Fc region. The *N*-glycan that is attached to the conserved asparagine (Asn) residue at position 297 in the Fc comprises a core structure of *N*-acetylglucosamine and mannose, plus additional carbohydrate residues, which can vary, including fucose, galactose, sialic acid and bisecting *N*-acetylglucosamine. (**B**) Scheme showing the described *N*-glycan in human IgG and activities of the enzymes studied in this work. The arrows indicate the carbohydrate units added or hydrolyzed by the corresponding enzymes. The human glycans are mainly classified as 'biantennary complex' structure with a core fucose and are often terminated with sialic acid. The third *N*-acetylglucosamine (GlcNAc) bisecting arm represents around 10% of human IgGs glycoforms. Only the α2-6-linked sialic acid is associated with the anti-inflammatory effects of IVIG. Black square, GlcNAc; white triangle, fucose; white circle, mannose; gray circle, galactose; black diamond, α2-6 linkage sialic acid. ST6Gal-I: β-galactoside α2-6 sialyltransferase-1, Neu1: neuramidase 1, β4GALT1: β1,4-galactosyltransferase 1, GLB1: β-galactosidase 1, FUT8: α1-6 fucosyltransferase , FUCA1:α-fucosidase.

Several mechanisms may account for the anti-inflammatory activity of IVIG in different disease states [5-8]. IgG binds to specific receptor molecules (FcγRs) through the Fc region with the *N*-linked complex biantennary glycan (*N*-glycan), which can be modified by specific carbohydrate moieties that have profound effects on binding affinity (Figure **1A**) [9]. IgG glycan modifications such as addition of sialic acid (Sia), galactose (Gal) or fucose, in serum IgG is associated with different inflammatory and immune system diseases, including rheumatoid arthritis and myasthenia gravis [10,11]. In mice, the addition of Sia to the non-reducing end of the *N*-glycan in IVIG leads to a reduction in systemic inflammation, although the exact mechanism is controversial [12-18]. Sialylated Fc structures are observed in 2 - 4% of IgG molecules in IVIG pooled from healthy adults [12,19-21]. Although sialylation of the penultimate Gal on the complex *N*-glycan can occur via either α2-3 or α2-6 linkage, only the α2-6-linked sialic acid (α2-6Sia) is associated with the anti-inflammatory effects of IVIG (Figure **1B**) [22,23]. 

β-Galactoside α2-6 sialyltransferase-I (ST6Gal-I) acting on the Gal(β4)GlcNAc terminal structure of the biantennary *N*-glycan of the Fc domain catalyzes the addition of α2-6Sias (Figure **1B**) [20]. Although the addition of α2-6Sias in IgG and the expression of ST6Gal-I, is thought to be critical for *in vivo* regulation of inflammation, none of these variant glycosylation patterns of IgG has been implicated in treatment response in KD [24-30]. Our first hypothesis was that IVIG preparations given to IVIG-resistant KD patients have lower levels of sialylation and thus limited anti-inflammatory effect. The second hypothesis was that IVIG-resistant KD patients have lower levels of sialylated endogenous IgG compared to IVIG-responsive patients. To investigate the mechanism of IVIG-response in KD patients, we measured levels of α2-6Sias, fucose, and Gal in IgG from IVIG-responsive and -resistant KD subjects. Both transcript and protein levels of ST6Gal-I were analyzed in whole blood and cell lysates from Epstein-Barr virus (EBV)-transformed B cell lines established from IVIG-responsive and -resistant subjects. 

## Materials and Methods

### Subjects

We selected 20 children (10 IVIG-resistant patients and 10 age-matched IVIG-responders) who met American Heart Association clinical criteria for complete or incomplete KD during their acute illness from among the subjects enrolled in a biobanking study [31]. All KD subjects were initially treated with IVIG (2 g/kg) and aspirin (80 mg/kg/day) during the acute phase. Clinical data included age, sex, illness day at diagnosis, CA status, ethnicity, and pre-treatment laboratory values (Table **1**). IVIG-resistance was defined as persistent or recrudescent fever (T ≥38.0 °C rectally or orally) at least 36 hours after the completion of the IVIG infusion. 

**Table 1:** Clinical characteristics and laboratory values at the acute time point for study subjects in glycosylation assays.

	**KD subjects**			**Control subjects**			
	**IVIG-responsive (n=10)**	**IVIG-resistant (n=10)**	**p**	**Febrile control (n=10)**	p^**^	**Healthy control (n=10)**	p^**^
Age at diagnosis, years	3.0 (2.3-3.2)^*^	2.3 (1.5-3.5)	NS	2.3 (1.1-3.3)	NS	2.4 (2.0-2.5)	NS
Male, n (%)	7 (70)	3 (30)	NS	8 (80)	NS	7 (70)	NS
***Illness day at sample collection, days	5.0 (5.0-6.0)	5.0 (4.3-6.8)	NS	4.0 (2.3-6.0)	NS	NA	
Incomplete KD, n (%)	1 (10)	1 (10)	NS	NA		NA	
Coronary artery aneurysms, n (%)	2 (20)	2 (20)	NS	NA		NA	
Ethnicity, n							
Asian	2	3		1		0	
African-American	1	0		0		0	
Caucasian	2	0		1		5	
Hispanic	3	3		5		5	
More than race	2	4		3		0	
CRP, mg/dl	6.3 (5.1-12.7)	11.8 (6.5-20.1)	NS	1.0 (2.2-2.4)	< 0.001	NA	
ESR, mm/h	57 (50-76)	64 (52-74)	NS	18 (10-31)	< 0.001	NA	
WBC, ×10^3^/mm^3^	10.5 (9.8-14.5)	14.5 (11.6-17.6)	NS	9.5 (7.5-14.8)	NS	NA	
Absolute neutrophil count, cells/mm^3^	7683 (4504-9579)	10208 (8256-3653)	NS	4670 (2738-8488)	NS	NA	
Platelet count, ×10^3^/mm^3^	323 (266-530)	351 (300-415)	NS	331 (256-357)	NS	NA	
ALT, IU/l	38 (23-66)	35 (30-103)	NS	30 (18-38)	NS	NA	
GGT, IU/l	43 (21-70)	41 (26-81)	NS	16 (14-21)	0.006	NA	

^*^ Values are presented as median (IQR). p-values were calculated by Mann–Whitney U test for continuous variables and by Fisher’s exact tests for categorical variables.

^**^ p-values are for comparisons between KD and control patients.

^***^ Illness day 1: first calendar day of fever.

KD: Kawasaki disease, IVIG: intravenous immunoglobulin, CRP: C-reactive protein (Normal range: <0.5 mg/dl) , ESR: erythrocyte sedimentation rate, WBC: white blood count, ALT: alanine aminotransferase (Normal range: 10-25 IU/L), GGT: gamma-glutamyltransferase (Normal range: 10-22 IU/L), NA: Not applicable, NS: Not Significant.

Ten age-similar subjects with other rash-fever illnesses and 10 age-similar healthy children served as controls (Table **1**). Febrile control subjects were previously healthy children recruited from Emergency Department at Rady Children’s Hospital San Diego who had a self-limited illness with at least 3 days of fever and at least one of the clinical signs of KD (rash, conjunctival injection, cervical lymphadenopathy, erythematous oral mucosa, and erythematous or edematous hands or feet) and were diagnosed as self-limited viral syndrome. Healthy subjects were children undergoing minor elective surgery for polydactyly. 

### Samples

Whole blood was collected from KD subjects before treatment with IVIG and one year later and from febrile subjects at the time of phlebotomy for diagnostic evaluation, and from healthy subjects at the time of preoperative venous cannulation. Serum was separated within 48 hours of collection and stored at -70°C until samples were analyzed. RNA was extracted using PAXgene blood miRNA kit (PreAnalytix, QIAGEN, NV) from whole blood collected in PAXgene tubes (Qiagen, Hilden, Germany). A 1 ml aliquot of IVIG administered to each KD subject was also collected.

### Purification of IgG

IgG from 500 μl of serum was isolated using a 0.2 mL NAb^TM^ protein A column (Thermo Scientific, MA) according to the manufacturer’s instructions. Briefly, serum was diluted in binding buffer (100 mM sodium phosphate containing 150 mM NaCl, pH 7.2) and passed through the column. IgG was eluted in 400 μl fractions using buffer (pH 2.8) provided into collection tubes preloaded with 40 μL of 1 M Tris-HCl 1 M, pH 8.5 for neutralization. IgG levels were measured using a NanoDrop^®^ ND-1000 Spectrophotometer (Thermo Scientific, MA). 

### Isolation of F(ab')_2_ and Fc fragments

After buffer was exchanged using Zeba™ Spin Desalting Columns (Thermo Scientific, MA) to 20 mM sodium phosphate/HCl containing 10 mM EDTA (pH 7.0), IgG samples (250 μg) were incubated with 50% immobilized papain (Thermo Scientific, MA) for 6 hours at 37 °C in 20 mM sodium phosphate/HCl containing 10 mM EDTA (pH 7.0). Papain-digested IgG was purified again using the NAb^TM^ protein A column as previously described. Specific cleavage of the hinge region of IgG, releasing F(ab')_2_ and Fc fragments, was verified by Western blot analysis. 

### Western blot analysis

Purified Fc and F(ab')_2_ fragments from IVIG, serum, or cell lysates were resolved on 12% polyacrylamide gels (Bio-Rad, CA) under reducing conditions using 98% 2-mercaptoethanol (Bio-Rad, CA). After electrophoresis, proteins were transferred onto PVDF (Bio-Rad, CA) membranes following standard procedures. For Sia detection, blocking with a Carbo-Free™ Blocking Solution (Vector Laboratories, CA), followed by incubation with either biotinylated Sambucus nigra agglutinin (SNA) lectin (2 μg/ml, B-1305, Vector Laboratories,CA) for 2-6 SA or biotinylated *Maackia amurensis* lectin-II (MAL-II) (4 μg/ml, B-1265, Vector Laboratories, CA) for α2-3 Sia linkage, followed by incubation with Streptavidin-Horseradish peroxidase (HRP) (DY991, R&D systems, MN). For detection of the Fc fragment of IgG or ST6Gal-I, membranes were blocked with a 5% BSA solution followed by incubation with primary antibodies (Mouse anti-human Fc antibody,1:5,000 dilution, MAB1304, Millipore, MA or Human ST6Gal-I antibody, 1:1:30,000 dilution, AF5924, R&D systems, MN) overnight at 4 °C, and detector antibodies (Sheep anti-mouse IgG ECL HRP-linked antibody, 1: 50,000 dilution, NA931 GE Healthcare, NJ or Biotinylated anti-rabbit IgG antibody:1:300,000dilution, NA934,GE Health care, NJ) for 2 hours at room temperature, followed by streptavidinHRP (R&D systems, MN) for 2 hours at room temperature. Detection was performed using the ECL Plus Western Blotting Detection Reagents (GE Health care, NJ) and exposure to X-ray film.

To quantitate the fraction of α2-6Sias on infused IVIG (5% or 10% Gammagard Liquid, Baxter, IL) and endogenous IgG from KD and control subjects, IgG was isolated using Protein A columns from the IVIG preparation infused into each KD subject and from the serum of 10 IVIG-responsive and 10 resistant KD subjects at the acute (pre-treatment) and convalescent (1 year after KD onset) time points, and from 10 age-similar febrile and 10 healthy control children (Table **1**). Median yields were 3.8 mg (IQR: 3.5-3.9 mg) and 1.9 mg (1.5-2.3 mg) of IgG from 500 μl of IVIG and serum, respectively. Following papain digestion, sodium dodecyl sulfate polyacrylamide gel electrophoresis (SDS-PAGE) showed Coomassie-stained bands of the expected sizes for Fc (28 kDa) and F(ab')_2_ (25 kDa) (Figure **S1A**). Western blotting for papain-digested IgG detected only α2-6Sias but not α2-3-linked sialic acid (α2-3Sias) on the Fc portion of IgG (Figure **S2B**). Neither α2-6Sias nor α2-3Sias was detected on the F(ab')_2_ portion. Therefore, whole IgG was used in the following assays to evaluate glycosylation of N-glycan on the Fc portion.

### Glycosylation assays (Figure 2)

**Figure 2:**
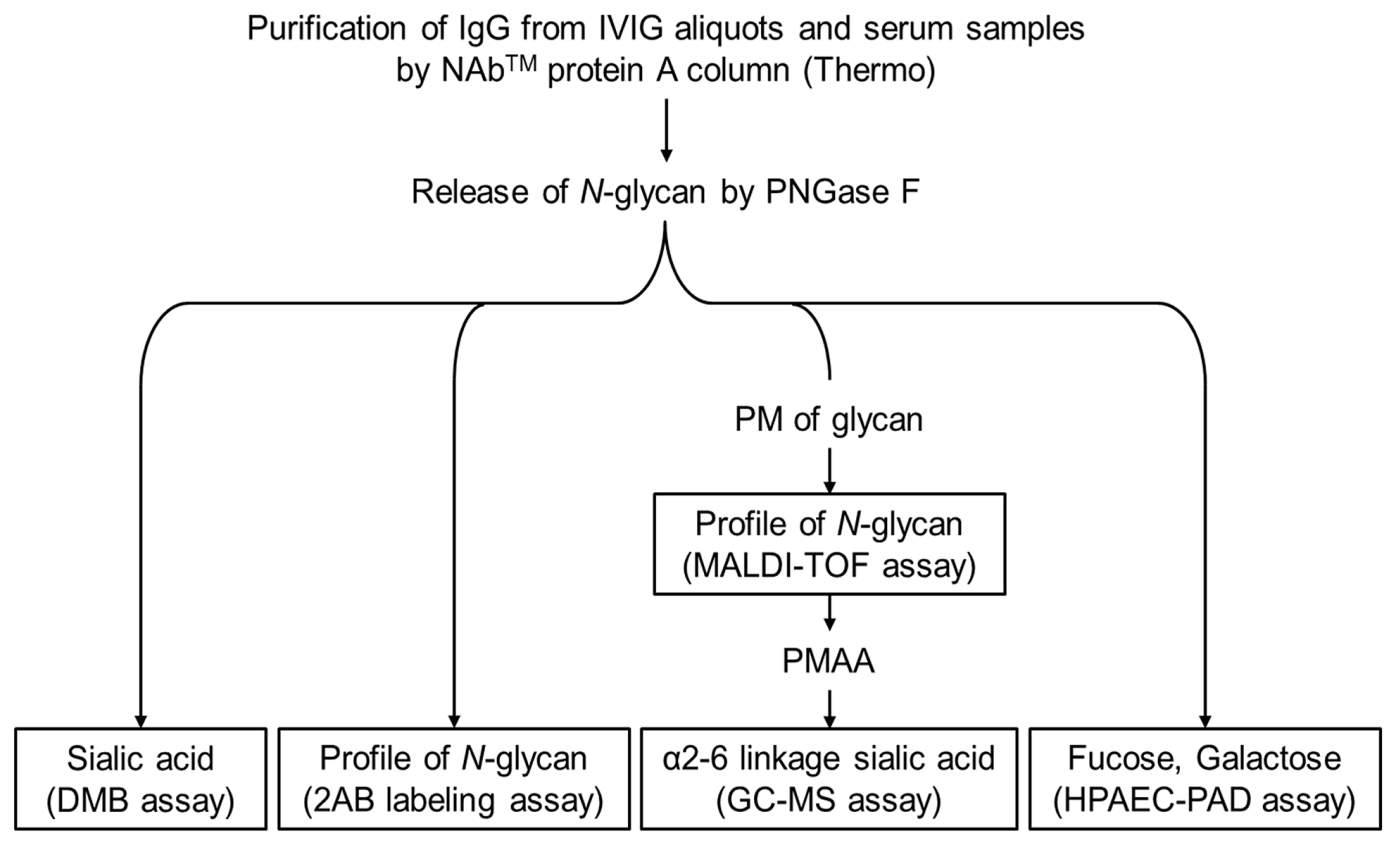
Overview of different glycosylation assays. PNGase F: peptide -N-Glycosidase F, DMB assay: 1,2-diamino-4,5-methylenoxybenzene (DMB)-labeled sialic acid by HPLC, 2AB: 2-aminobenzamide, PM: per-O-methylation, MALDI-TOF: matrix-assisted laser desorption-ionization time-of-flight. PMAA: partially methylated alditol acetate, GC-MS: gas chromatography–mass spectrometry, HPAEC-PAD: high performance anion exchange chromatography with pulsed amperometric detection.

#### Release of N-glycan from purified IgG

250 μg of IgG was solubilized and denatured using denaturing buffer containing 0.5% Sodium Dodecyl Sulfate (SDS) and 2-Mercaptoethanol. Fivefold excess NP-40 relative to SDS was added and the reaction mixture was kept at room temperature for 1h. Then *N*-glycans were released using PNGaseF from the denatured IgG. The enzymatic digestion was done for 24h at 37 °C incubator to ensure complete release of the *N*-glycans. Released *N*-glycans were purified by passing the reaction mixture over Sep-Pak C18 cartridge (Waters, MA) followed by a porous graphitized carbon (PGC) cartridge (Thermo Scientific, MA). Under aqueous conditions, oligosaccharides bind to the PGC cartridge, while salts and buffers pass through. *N*-glycans were then eluted with aqueous 30% acetonitrile containing 0.1% Trifluoroacetic acid (TFA). Acetonitrile and TFA were removed by lyophilization and purified *N*-glycans were used for subsequent analyses. 

#### Quantitation of Sia after flourophore tagging using the reverse phase (RP) high-performance liquid chromatography (HPLC)

*N*-glycans isolated from 10 μg IgG were reacted with 2 M Acetic Acid at 80°C for 3 hours to release Sia from *N*- glycans. The released Sias were purified by ultrafiltration through a 3,000 dal Molecular weight cut-off spin filter (Millipore, MA) and tagged with 1,2-diamino-4,5-methylenedioxybenzene (DMB)-labeled Sia. The DMB-tagged Sias were analyzed by RP-HPLC using C18 column (Acclaim 120, Thermo Scientific, MA, 4.6 x 250mm, 5um particle size) with on-line fluorescence detection (Dionex RF2000, Dionex, CA). 

#### Monosaccharide analysis by the high performance anion exchange chromatography with pulsed amperometric detection (HPAEC-PAD)

*N*-glycans from 10 μg IgG were treated with 200 μl of 2 M TFA at 100 °C for 4 hours to cleave all glycosidic linkages. Acid was evaporated by dry nitrogen flush and co-evaporation twice with 1:1 isopropanol to ensure complete removal of acid. Finally, the samples were dissolved in water and analyzed by HPAEC-PAD using a CarboPac PA-1 column (Dionex, CA). The monosaccharides from the *N*-glycans were quantified using known quantities of authentic monosaccharide standards.

#### 2AB labeling of glycans

The 2-AB was reductively coupled with purified (I think it was mentioned in the scheme) *N*-glycans with a free reducing end. In brief, 6.0 mg of 2-AB reagent was dissolved in 200 μl of 35:65 glacial acetic acid: DMSO mixture. The entire mixture was added to 6.5 mg of sodium cyanoborohydride in a separate tube. The reaction mixture was mixed thoroughly by vortexing and sonication. 10 μl of this tagging reagent was added to each sample and incubated at 65 °C for 2.5 hours. After 2AB labeling, the samples were purified by passing through over a Glycoclean S-cartridge and dried on a Speed-vac before further analysis.

#### Per-O-methylation (PM) of glycans

*N*-glycans from 200 μg IgG were dissolved in dry DMSO and stirred for one hour until the sample was completely dissolved. Sodium hydroxide slurry in DMSO was added as a reacting base followed by addition of 200 μl of methyl iodide. The reaction mixture was vigorously stirred for 1 hour followed by addition of another aliquot of 100 μl of methyl iodide with continued stirring for additional 30 minutes. The reaction was stopped by adding 1 ml of ice-cold MilliQ-water followed by addition of 1 ml of chloroform. The reaction mixture was then vortexed and centrifuged at 2000 rpm for 2 minutes to separate the chloroform and aqueous layers. The aqueous layer was discarded and the chloroform layer was washed three times with 1 ml of water. The chloroform layer containing the partially methylated glycans was then dried and used for further analysis by the matrix-assisted laser desorption-ionization time-of-flight mass spectrometry (MALDI-TOF MS) or as partially methylated alditol acetate (PMAA) derivative for linkage analysis by the gas chromatography-mass spectrometry.

#### MALDI-TOF MS profiling of glycans

Permethylated glycans were dissolved in 20 ul of absolute methanol and mixed with SDHB matrix in a 1:1 ratio and spotted onto the MALDI plate. The samples were air-dried and analyzed in positive reflectron mode. 

#### PMAA

The PM samples were hydrolyzed by 4N TFA at 100 °C for 4 hours and acid was removed by co-evaporation using 1:1 isopropanol: water (v/v) mixture under dry nitrogen flush followed by removal of the acid using a dry nitrogen flush. The acid was removed completely by coevaporation twice using 1:1 isopropanol: water mixture under dry nitrogen flush. Hydrolyzed samples were reduced overnight at room temperature using sodium borodeuteride in 1 M ammonium hydroxide solution. Excess reducing agent was neutralized on an ice bath by slowly adding ice-cold 30% acetic acid. The boric acid thus formed was removed by repeated co-evaporation using 200 μl of acidified methanol thrice and absolute methanol thrice sequentially. Dried samples were stored on a vacuum desiccator on over P_2_O_5_ as desiccant for two hours and finally treated with 1:1 acetic anhydride: pyridine mixture at 100 °C for 1hour. Unreacted pyridine and acetic anhydride was removed by nitrogen flush and PMAA samples were dissolved in dichloromethane and injected in the gas chromatography-mass spectrometry (GC-MS).

#### GC-MS analysis of PMAA derivative (linkage analysis)

PMAA analysis was done by GC-MS using Restek-5 ms capillary column (30 m x 0.25 mm l x i.d., Restek, PA). Identification was achieved by using a combination of retention times (as compared to those of known standards analyzed under the same conditions) and mass fragmentation pattern. The results were shown by the ratio of the amounts of 6-linked Gal and inositol as the 6-linked Gal indicates the binding site of α2-6Sias. 

### Microarray Analysis of ST6GAL1

Two different microarray platforms (Illmina array and Affymetrix Human Genome U133 Plus 2.0 Array) were used to study two independent cohorts of KD subjects and the data were mined for the purpose of this study [32]. On the Illumina microarray, we analyzed whole blood transcript levels from 110 IVIG-responsive and 30 IVIG-resistant mixed ethnicity subjects at the acute (pre-treatment) and convalescent (at least three weeks after treatment) time points. The dataset was then normalized using quartile normalization procedure using PreprocessCore package for R. Finally log 2 transformation was performed. On the Affymetrix microarray we analyzed six IVIG-responsive and six -resistant Japanese subjects at the acute and convalescent (36 hours after end of treatment) time points [32]. 

### Quantitative (q)RT-PCR assay of ST6GAL1

To validate the microarray data, we performed qRT-PCR for *ST6GAL1* using whole blood RNA from the cohort of 20 KD subjects (10 IVIG-responsive and 10 -resistant subjects) whose IgG sialylation levels had been previously measured (Table **1**). We evaluated *ST6GAL1* transcript levels using four primers for the conserved region (Exon VI), Transcript 1-specific region (P1 promoter region on Exon I, highly expressed on hepatocytes), Transcript 2-specific region (Exon X-I region, highly expressed in mature B-cells, kidney, and spleen) and Transcript 3-specific region (Exon Y-Z region, expressed in many different cells) on *ST6GAL1* mRNA (Figure **S2**) [33,34]. 

Twenty KD subjects with paired samples (acute and 1 year after onset) and 10 febrile control (viral syndrome) samples, whose IgG has been analyzed for sialylation, were analyzed for *ST6GAL1* transcription levels. qRT-PCR was performed as previously described using commercially available Taqman primers for *ST6GAL1* (conserved: Hs00949383_m1; transcript1: AILJJB5; transcript 2: AI6RN2A; and transcript 3: Hs00415704_m1, TaqMan, Life Technologies, NY) and primers designed to detect specifically transcript 1 and 2; transcript 1: (forward primer: 5′-CTGTCTCTTATTTTTTGCCTTTGCAG-3′, reverse primer: 5′-TCCATGGGAGGGAAGGTTTATT-3′; probe: 5′-ATCCTGAGAAAAATGG-3′), and transcript 2 (forward primer: 5′-TCTGCAGCATCCTTGATGATAAA-3′, reverse primer: 5′-GCCAAGGCCCATTTTTCTC-3′, probe: 5′-AATATGAGTTTTGATCATCC-3′) [35]. Relative abundance of the target transcripts was normalized to the expression level of the housekeeping gene, TATA box-binding protein-associated factor, RNA polymerase I, B (*TAF1B*) (Hs01057259-m1, Applied Biosystems), as previously described [36].

### Isolation of B cells from subjects and incubation of EBV- transformed B cell lines

Peripheral blood mononuclear cells (PBMC) were isolated from heparinized blood samples by gradient centrifugation with Ficoll Hypaque (Sigma-Aldrich, MO). B cell lines were created by infecting PBMC with supernatant from an EBV-producing marmoset-derived cell line B95-8 (American Type Culture Collection, VA) and expanded in complete RPMI supplemented with 10% fetal calf serum (HyClone Laboratories, UT).

### Cell lysis

Cells were harvested and lysed in 300 mM NaCl, 50 mM Tris, pH 7.4 contain 0.5 % Triton X-100 and cOmplete anti-protease cocktail (Roche, Basel). Protein concentrations were quantified using the NanoDrop® ND-1000 Spectrophotometer (Thermo Scientific, MA).

### ELISA assay for ST6Gal-I

ST6Gal-I in cell lysates, culture supernatants, serum, and plasma were measured using the human ST6Gal-I ELISA Kit (MyBioSource, CA) according to the manufacturer’s instructions.

### Statistical analysis

Data were analyzed using GraphPad Prism (GraphPad Software, Inc., La Jolla, CA) software, and presented as medians and IQR for continuous variables and frequency counts and percentages for categorical variables. Univariate analysis was performed with Fisher’s exact tests for categorical variables and Wilcoxon rank sum tests for continuous variables. Paired data for acute and convalescent samples were analyzed using a Wilcoxon signed rank test. A *p*-value < 0.05 was considered significant. 

### Study approval

The Human Research Protection Program of the University of California, San Diego approved this research protocol and written informed consent was obtained from the parents of all subjects and adolescent or child assent was obtained as appropriate. The study protocol conforms to the ethical guidelines of the 1975 Declaration of Helsinki.

## Results

### Sialylation of endogenous IgG and infused IVIG

**Figure 3:**
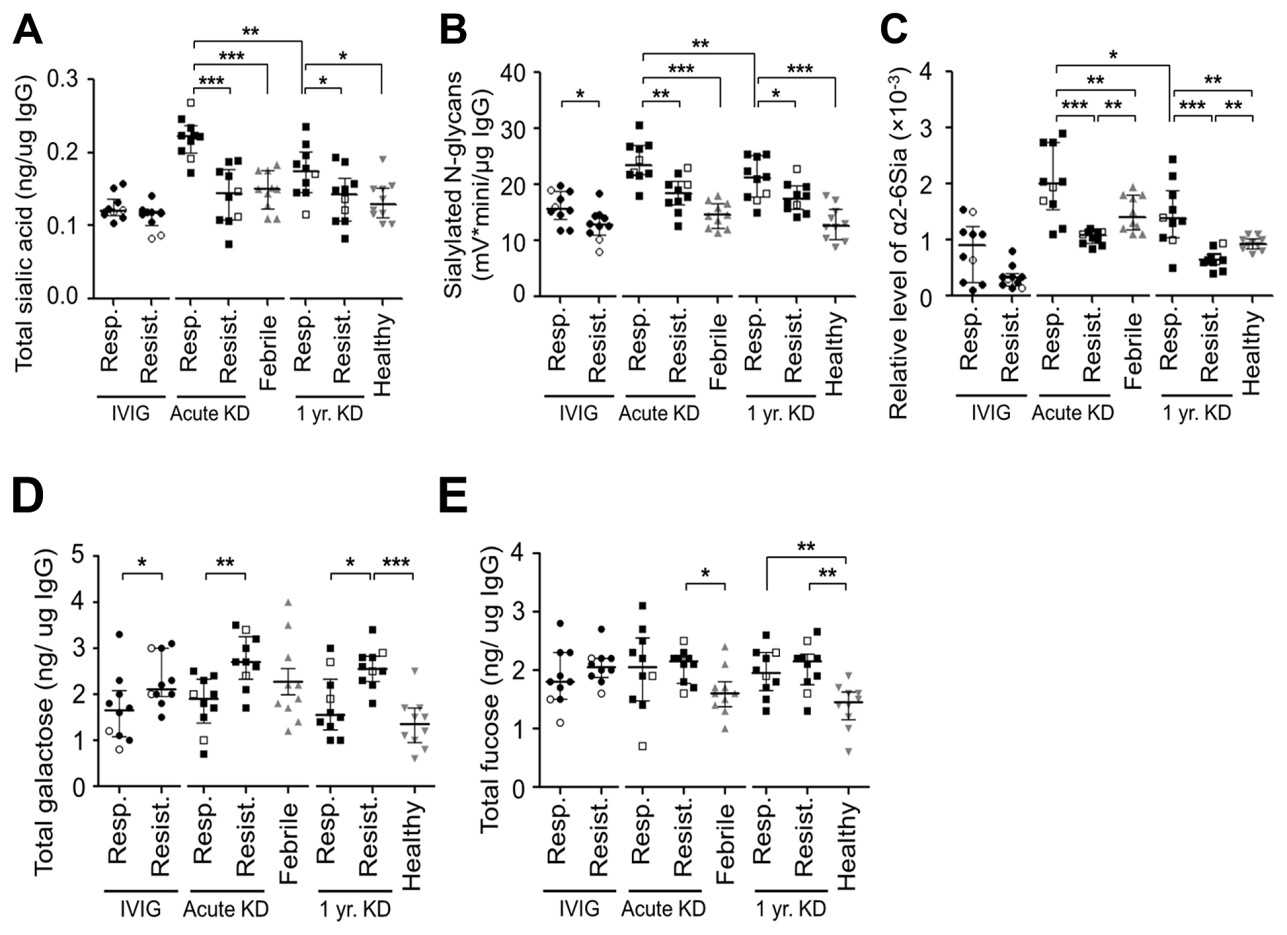
Levels of sialic acid, fucose, and galactose in infused IVIG and endogenous IgG from IVIG-responsive and -resistant KD subjects at acute and 1 year time points using four different assays. (**A**) Levels of total sialic acid in IVIG and endogenous IgG, measured by DMB assay, (**B**) Levels of sialylated *N*-glycans in IVIG and endogenous IgG, measured by 2AB-labeling assay, (**C**) Levels of α2-6 linked sialic acid relative to 5 μg inositol in IVIG and endogenous IgG measured by GC-MS assay. (**D**) Galactose levels on IVIG and endogenous IgG measured by HPAEC-PAD assay. (**E**) Fucose levels on IVIG and endogenous IgG measured by HPAEC-PAD assay. The same subjects were used for all assays. Open symbols: Subjects with coronary aneurysms. Results shown as medians and IQR. p-values by Mann-Whitney U test. *p<0.05, **p<0.01, ***p<0.001. IVIG: intravenous immunoglobulin, KD: Kawasaki disease, DMB assay: 1,2-diamino-4,5-methylenoxybenzene (DMB)-labeled sialic acid by HPLC, 2AB: 2-aminobenzamide, GC-MS: gas chromatography–mass spectrometry. HPAEC-PAD: high performance anion exchange chromatography with pulsed amperometric detection.

Sia levels in endogenous IgG from IVIG-responsive and -resistant KD and control subjects were measured using three complementary techniques (Figure **2**). We first quantified the levels of total Sia using a semi-quantitative analysis of 1,2-diamino-4,5-methylenedioxybenzene (DMB)-labeled Sia with the reverse phase (RP) high-performance liquid chromatography (HPLC) (Figure **3A** and Table **S1**). Total Sia levels in endogenous IgG were significantly lower in IVIG-resistant KD subjects than in IVIG-responsive KD subjects at the acute and one-year time points (Acute: p <0.001, and one-year: p =0.014). Total Sia levels in endogenous IgG from IVIG-responsive KD subjects were significantly higher during the acute phase than at one year after KD onset, which we presumed to represent their healthy baseline levels (p =0.002). In contrast, the total Sia levels on endogenous IgG from IVIG-resistant subjects were equally low at the acute and one year time points. 

As a second approach, levels of different glycan structures, including sialylated *N*-glycans, released from IgG were analyzed using the 2-aminobenzamide (2AB) labeling assay (Figure **3B** and Table **S1**). In keeping with the results from the DMB assay, the levels of sialylated *N*-glycans in endogenous IgG from IVIG-resistant subjects were significantly lower than those in IVIG-responsive subjects at both the acute and one year time points (Acute: p =0.002, and one-year: p =0.039). Structural analysis by the 2AB assay was validated by the matrix-assisted laser desorption-ionization time-of-flight mass spectrometry (MALDI-TOF MS) analysis of per-methylated *N*-glycans (Figure **S3**). 

The third approach quantified the levels of α2-6Sias in endogenous IgG from IVIG-responsive and -resistant KD subjects by the gas chromatography-mass spectrometry (GC-MS) (Figure **3C** and Table **S1**). Consistent with previous results, IVIG-responsive subjects had significantly higher α2-6Sias compared to IVIG-resistant subjects at both the acute and one-year time points (p <0.001, respectively) and had higher levels during the acute phase compared to one year later (p =0.014). 

Thus, the three analyses above consistently demonstrated significant differences between endogenous IgG from IVIG-responsive and -resistant subjects irrespective of time points tested. No relationship was found between Sia levels and the occurrence of CA aneurysms in any of the three assays. All three assays demonstrated higher Sia levels in endogenous IgG in IVIG-responsive subjects (Figure **3A-C**). 

Sia levels in therapeutic IVIG were also assessed using these three methodologies. Only the assay for sialylated *N*-glycans showed a difference in the levels between IVIG administered to responsive compared to resistant subjects (p =0.019) (Figure **3A-C** and Table **S1**). As expected, the levels of Sia in the infused IVIG were significantly lower in all three analyses compared to the endogenous IgG from KD subjects. 

### Terminal galactose and fucose in endogenous IgG and infused IVIG

Levels of Gal in endogenous IgG from IVIG-resistant subjects were significantly higher than in IVIG-responsive subjects at the acute and one-year time points (Acute: p =0.004, and 1 year: p =0.016) (Figure **3D**). Total Gal levels on infused IVIG, quantified by high performance anion exchange chromatography with pulsed amperometric detection (HPAEC-PAD), were also significantly higher in IVIG-resistant subjects compared to IVIG-responsive subjects (median 1.6 and 2.1 ng/μg IgG for IVIG-responsive and -resistant subjects, respectively, p =0.048). 

The HPAEC-PAD assay revealed no significant differences in total fucose in infused IVIG or endogenous IgG, between IVIG-responsive and -resistant subjects at either time point. However, endogenous IgG fucose levels were higher among IVIG-resistant KD subjects compared to febrile controls acutely (p =0.014) and higher among IVIG-responsive and -resistant KD subjects compared to healthy control subjects at the one-year time point (IVIG-responsive: p =0.008, and -resistant subjects: p =0.004) (Figure **3E**). 

### Microarray analysis of transcript levels of glycosylation enzymes

To assess the role of enzymes in glycosylation, we analyzed the transcript levels of enzymes involved in α2-6-linked sialylation, galactosylation, and fucosylation including *ST6GAL1* and neuraminidase 1 (*NEU1*) for sialic acid, β1-4 galactosyltransferase-I (*B4GALT1*) and β-galactosidase-I (*GLB1*) for Gal, and α1-6 fucosyltransferase (*FUT8*) and α-fucosidase (*FUCA1*) for fucose, by making use of our KD cDNA microarray datasets reported previously (Table **2** and Figure **1B**)[32]. 

**Table 2:** Transcript levels of glycosylation enzymes in microarray data sets on different platforms.

			**Acute/Convalescent**		**IVIG-responsive /-resistant subjects at each time point**			
					**Acute**		**Convalescent**	
**Array**	**Symbol**	**Probe ID**	**Fold-difference**	**p**	**Fold- difference**	**p**	**Fold-difference**	**p**
**Illumina** (n: 110 IVIG-responsive, 30 IVIG-resistant subjects)	*ST6GAL1*	1260601	0.62	< 0.001	1.43	0.002	1.36	0.004
		1710630	0.77	< 0.001	1.06	NS	0.98	NS
	*NEU1*	4200692	1.76	< 0.001	0.93	NS	1.13	NS
	*B4GALT1*	3460386	1.11	< 0.001	0.91	0.024	0.92	NS
	*GLB1*	1340634	1.37	< 0.001	1.10	NS	0.94	NS
		4560064	1.43	< 0.001	1.04	NS	0.63	NS
	*FUT8*	3870497	0.77	< 0.001	1.29	NS	0.95	NS
	*FUCA1*	2060121	1.04	NS	0.96	NS	0.92	NS
**Affymetrix** (n: 6 IVIG-responsive, 6 IVIG-resistant subjects)	*ST6GAL1*	201998_at	0.56	0.025	1.95	0.009	1.69	0.015
	*NEU1*	208926_at	1.32	0.031	0.74	NS	0.59	NS
	*B4GALT1*	201883_s_at	1.01	NS	1.14	NS	0.86	NS
		228498_at	1.26	NS	0.73	NS	0.79	NS
		238987_at	0.99	NS	0.90	NS	0.87	NS
	*GLB1*	201576_at	1.16	NS	1.02	NS	-1.04	NS
	*FUT8*	1554930_a_at	0.59	NS	0.85	NS	0.82	NS
		203988_s_at	0.64	NS	0.92	NS	0.65	NS
	*FUCA1*	202838_at	0.66	NS	1.10	NS	1.33	0.023

Nominal p-values were calculated by Mann–Whitney U test. NS: Not Significant. *ST6GAL1*: β-galactoside alpha-2,6-sialyltransferase 1, *NEU1*: neuraminidase 1, *B4GALT1*: β-1,4-galactosyltransferase 1, *GLB1*: β-galactosidase 1, *FUT8*: α-(1,6)-fucosyltransferase, *FUCA1*: α-fucosidase

Because the convalescent RNA samples were obtained at a point in time when the subjects were completely healthy, we assumed that the convalescent transcript levels represented the subject’s normal baseline. Of the 6 enzymes on the arrays, all except *FUCA1* were differentially expressed at acute and convalescent time points (Table **2**). 

**Figure 4:**
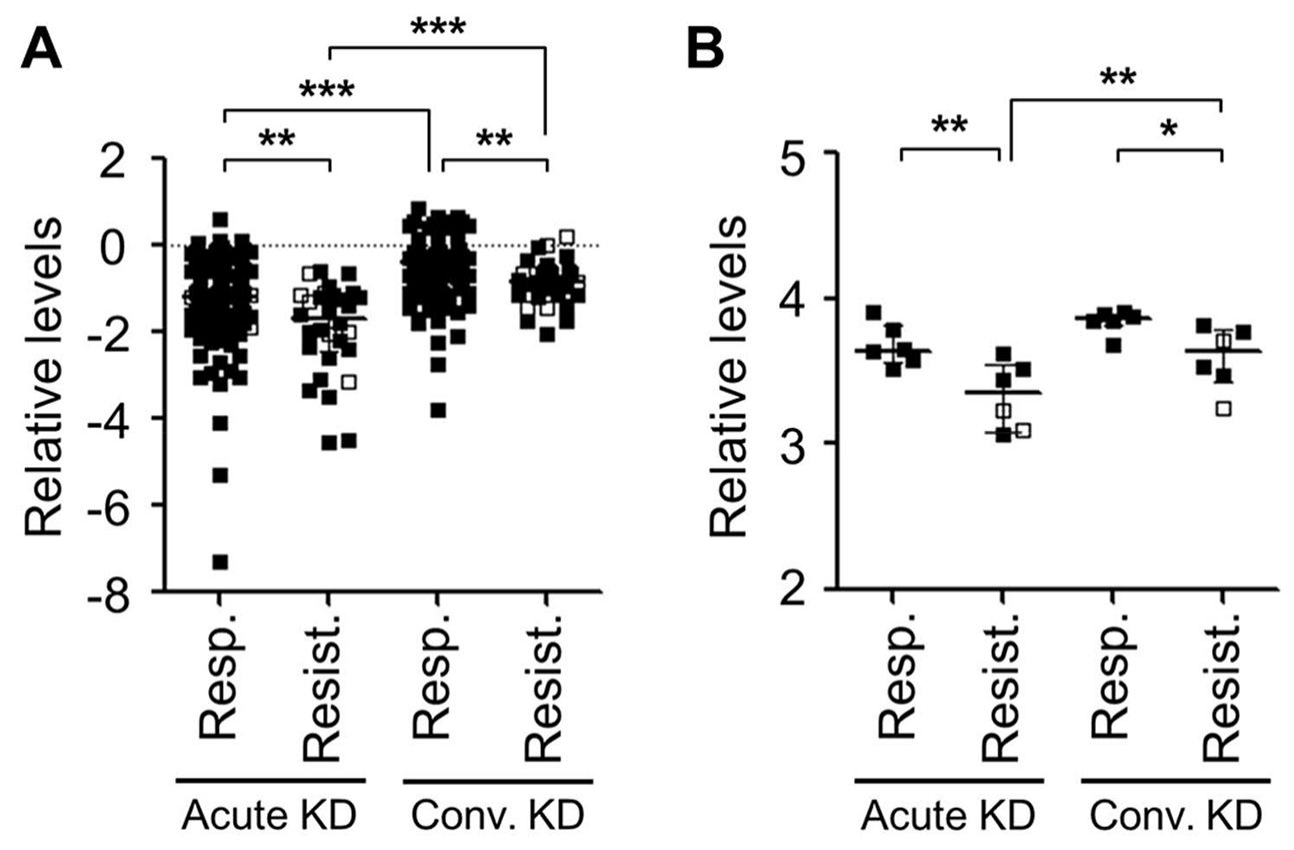
Microarray analysis of ST6GAL1 transcript. (**A**) Illumina array (probe ID: 1260601). On this microarray, whole blood transcript levels from 110 IVIG-responsive and 30 IVIG-resistant subjects at the acute (pre-treatment) and convalescent (at least 3 weeks after treatment) time points were analyzed. (**B**) Affymetrix Human Genome U133 Plus 2.0 Array (probe ID: 201998_at), whole blood transcript levels from six IVIG-responsive and six -resistant subjects (all Japanese) at the acute and convalescent (36 hours after end of treatment) time points were analyzed. Microarray clones were from exon VI (conserved region) of ST6GAL1 mRNA. Open symbols: Subjects with coronary aneurysms. Results shown as medians and IQR. p-values by Mann-Whitney U test. *p<0.05, **p<0.01, ***p<0.001. KD: Kawasaki disease.

A probe from the conserved exon VI of *ST6GAL1* from the Illumina array showed significantly lower transcript levels in IVIG-resistant subjects compared to IVIG-responsive subjects at both the acute and convalescent time points (Acute: p = 0.002, and Convalescent: p = 0.004) (Figure **4A** and Table **2**). On the Affymetrix array, the transcript levels of *ST6GAL1* from exon VI in IVIG-resistant subjects were also significantly lower than in IVIG-responsive subjects at both time points (Acute: p = 0.009, and Convalescent: p = 0.015) (Figure **4B** and Table **2**). Transcript levels for *NEU1* were up-regulated in all acute KD subjects compared to the convalescent time point (Illumina: p <0.001, Affymetrix: p =0.031). No differences were seen for *NEU1* between IVIG-responsive and -resistant KD subjects on these platforms.

Because there were significant differences in IgG fucosylation between KD and healthy control subjects, we analyzed *FUT8* and *FUCA1* transcript levels. The only significant difference was between the responsive and resistant subjects at the convalescent time point (Illumina: p <0.001) (Table **2**).

### Differential expression of *ST6GAL1* by qRT-PCR

**Figure 5:**
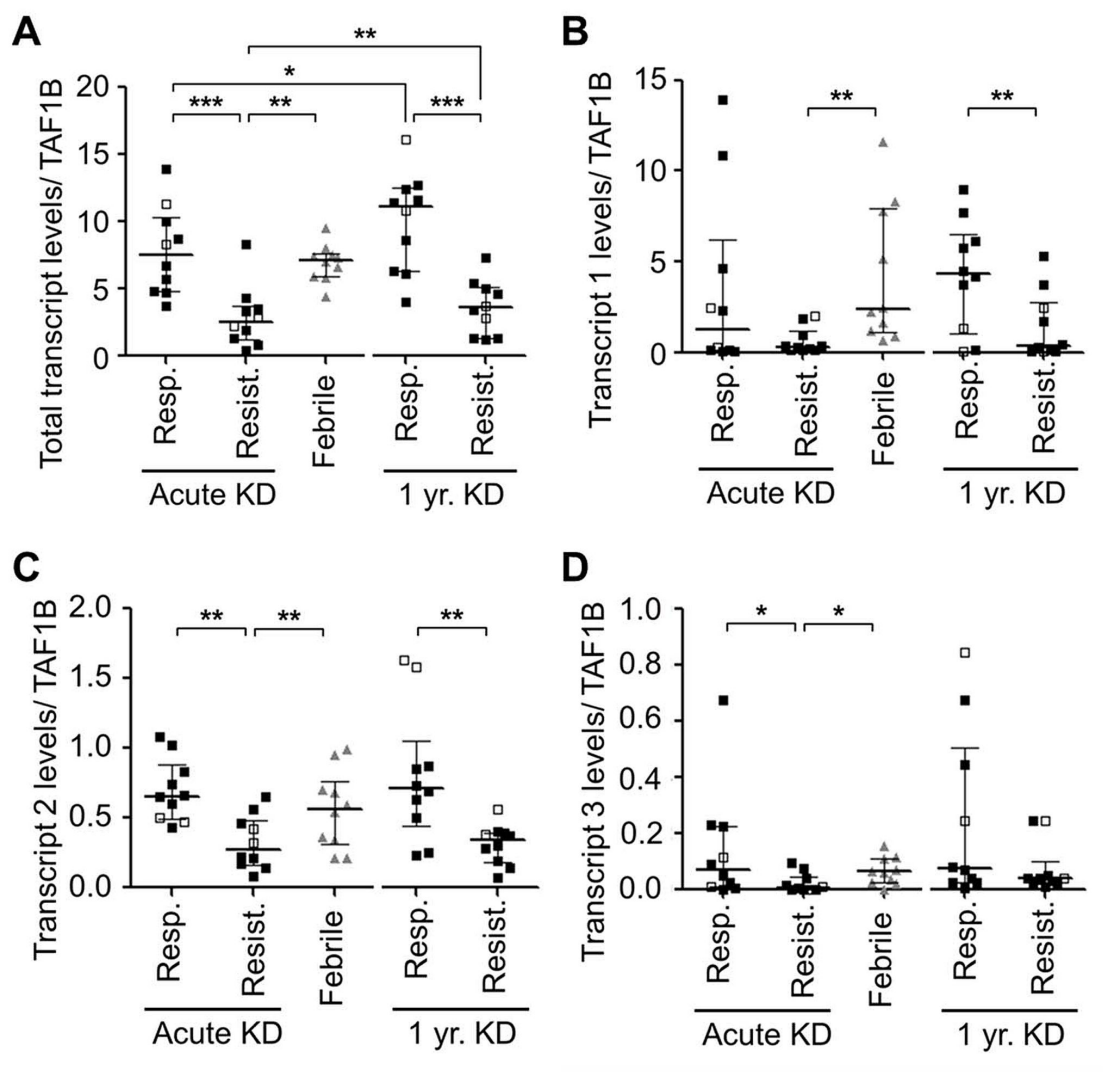
Whole blood transcript levels of ST6GAL1 in KD patients using qRT-PCR. Transcript levels of ST6GAL1 were analyzed with 4 different primer pairs. (**A**) Total ST6GAL1 transcript levels amplified with primers for conserved region spanning exons V and VI, (**B**) ST6GAL1 Transcript 1 levels encoded by the P1 promoter (hepatocytes). (**C**) ST6GAL1 Transcript 2 levels amplified with primer encoding exons X and I (B cells) (**D**) ST6GAL1 Transcript 3 levels encoding exons Y and Z (all cells). Results are relative units of ST6GAL1 transcripts normalized for expression of housekeeping gene, TAF1B. Open symbols: Subjects with coronary aneurysms. Bars are medians and IQR. p-values by Mann-Whitney U test. *p<0.05, **p<0.01, ***p<0.001. KD: Kawasaki disease.

We evaluated *ST6GAL1* transcript levels using four different primer pairs corresponding to a conserved region (Exons V-VI) and three unique transcripts: Transcript 1-specific region (Exon I, highly expressed in hepatocytes), Transcript 2-specific region (Exons X-I, expressed in mature B-cells, kidney and spleen) and Transcript 3-specific region (Exons Y-Z region, expressed in many different cells) Figure **S2**) [33,34]. The transcript levels of the conserved region and Transcript 2 (mature B cell-specific) were significantly lower in IVIG-resistant compared to IVIG-responsive subjects at both the acute and 1 year time points (Figure **5A and C**). *ST6GAL1* Transcript 1, which is initiated by the P1 promoter in hepatocytes, was significantly lower in IVIG-resistant subjects only at the one-year time point (p =0.034) (Figure **5B**). *ST6GAL1* Transcript 3 levels were low in all groups tested (p =0.043) (Figure **5D**). No correlation was observed between α2-6Sias levels on endogenous IgG and *ST6GAL1* transcript abundance levels (Figure **S4**). These results suggest that the difference between IVIG-responsive and resistant subjects was due to reduced levels of *ST6GAL1* Transcript 2, which is transcribed specifically in B cells, kidney, and spleen [34].

### *ST6GAL1* transcript and protein levels in B cell lines

**Figure 6:**
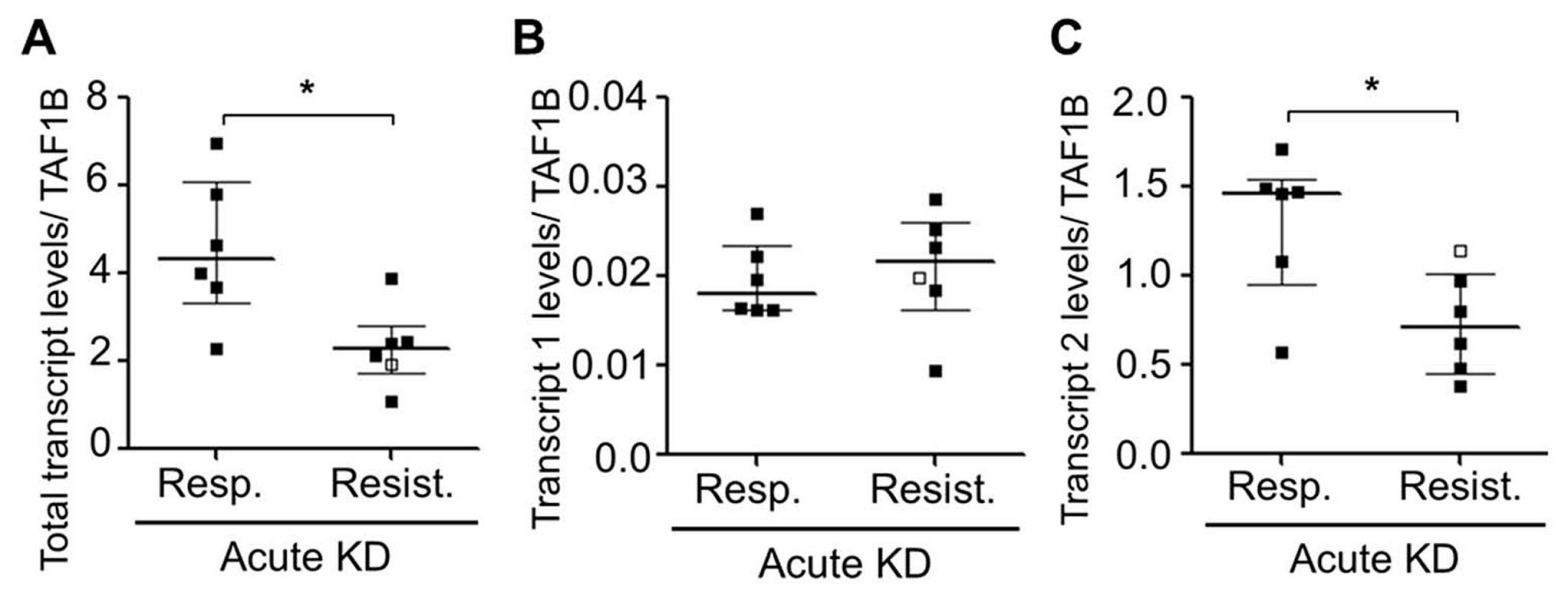
Expression transcript levels of ST6GAL1 in EBV-transformed B cell lines from IVIG-responsive and -resistant KD subjects at acute phase by qRT-PCR. (**A**) Amplification with primers for the conserved region in exon VI in ST6GAL1 mRNA. (**B**) Amplification with primers for Transcript 1 initiated from the P1 promoter in ST6GAL1 (P1 promoter and exon I region). (**C**) Amplification with primers for Transcript 2 (exon X and I region) in ST6GAL1. qRT-PCR results are presented as relative units of ST6GAL1 amplicon normalized for the housekeeping gene TAF1B. Open symbols: Subjects with coronary aneurysms. Results are shown as medians and IQR. p-values by Mann-Whitney U test. *p<0.05. KD: Kawasaki disease.

We measured *ST6GAL1* transcript levels in cell lysates from B-cell lines from six IVIG-responsive and six resistant acute KD subjects for whom lines were available (Table **S2**). Levels of transcripts containing the conserved region in exon VI and Transcript 2 were significantly lower in IVIG-resistant subjects compared to responsive subjects (Figure **6A and C**). Transcript 1 levels were not significantly different (Figure **6B**). Soluble (s)ST6Gal-I was present at low levels in the supernatant of B cell lines from the three IVIG-responsive subjects, while none was detected in the supernatants from resistant subjects (Figure **S5**). 

### Soluble ST6Gal-I in blood

**Figure 7:**
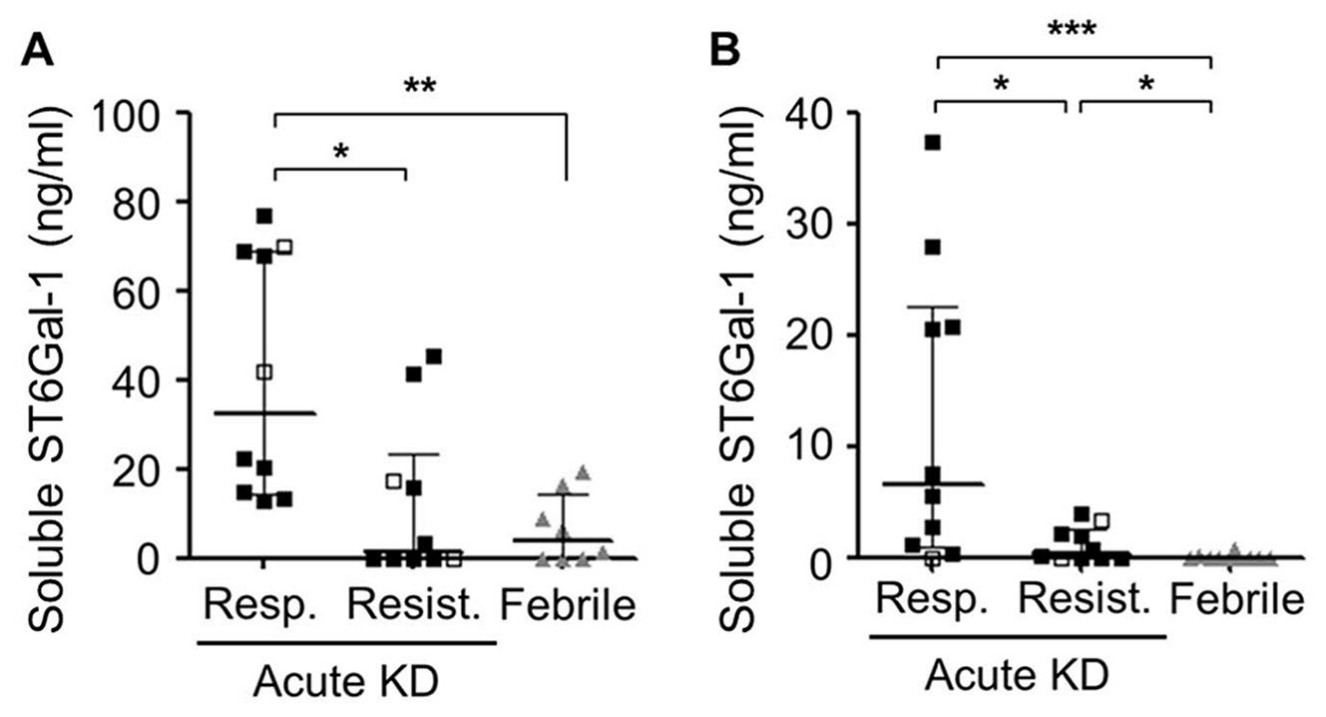
Soluble (s)ST6Gal-I in KD and febrile control subjects measured by ELISA assay. (**A**) sST6Gal-I in serum from IVIG-responsive and IVIG-resistant KD subjects and febrile controls. (**B**) sST6Gal-I levels in plasma from independent cohort of IVIG-responsive and IVIG-resistant KD subjects and febrile controls. Open symbols: Subjects with coronary aneurysms. Results shown as medians and IQR. p-values by Mann-Whitney U test. *p<0.05, **p<0.01, ***p<0.001. KD: Kawasaki disease.

Among the 20 KD subjects whose pre-treatment IgG sialylation was measured, serum sST6Gal-I levels of were significantly lower in IVIG-resistant subjects and febrile controls compared to IVIG-responsive subjects (p =0.013) (Figure **7A**). However, no correlation was observed between sST6Gal-I levels and *ST6GAL1* transcript levels in whole blood from KD subjects (data not shown). Using independent KD and febrile control cohorts (Table **S3**), IVIG-resistant subjects had significantly lower plasma sST6Gal-I levels compared to IVIG-responsive subjects(p =0.025) (Figure **7B**). sST6Gal-I levels did not distinguish the seven KD subjects with CA aneurysms from those with normal CA. 

## Discussion

In the first study to examine glycosylation patterns on endogenous IgG and infused IVIG in KD subjects, we found a consistent relationship between levels of sialic acid on endogenous IgG and response to treatment, with lower levels found in IVIG-resistant subjects (Figure **3A-C**). Furthermore, whole blood and B-cell line transcript levels and serum and plasma levels of sST6Gal-I, the enzyme responsible for adding the terminal sialic acid into the *N*-glycan structure of the Fc region, were significantly lower in IVIG-resistant subjects (Figure **4**-**7**). This finding persisted at one year and suggests a genetic difference in ST6Gal-I activity among children with acute KD resistant to IVIG. In contrast, the low levels of α2-6Sias in the infused IVIG did not appear related to the subject’s response to IVIG therapy. 

The glycans that decorate ligands and receptors play a critical role in modulation of the immune response. The *N*-linked oligosaccharide in the IgG Fc region maintains the steric structure of the two heavy chains in an open confirmation that is required for interaction with FcγRs expressed on different populations of immune cells, and removal of this glycan alters IgG binding affinity to FcγRs (Figure **1A**) [37,38]. Significant structural heterogeneity exists for the glycan profiles and composition in healthy adult populations, including the addition of fucose to the *N*-acetylglucosamine (GlcNAc) at the reducing ends, the addition of GlcNAc to the mannose between the binatennary structures (bisecting GlcNAc), or the addition of Gal and sialic acid at the non-reducing ends of the biantennary structure (Figure **1B**) [19,39]. Human IgG Fc regions with a sialylated, galactosylated, or fucosylated glycan have reduced affinities for FcγRs compared with asialylalted, agalactosylated, afucosylated Fc regions [40-44].

In this study, we confined our analysis to the sialylation of IgG. The negative charge of terminal sialic acid can alter protein function, affecting the binding and transport of ions and stabilizing the conformation of many proteins including enzymes [45]. Sialic acids in oligosaccharides regulate the affinity of receptors for these ligands and modulate processes involved in transmembrane signaling, fertilization, growth and differentiation [46]. Some research reports suggest that sialylated IgG induces anti-inflammatory signaling in immune cells via specific lectins such as the C-type lectin dendritic cell-specific intercellular adhesion molecule-3-grabbing non-integrin (DC-SIGN), CD22/siglec-2, and CD23, although this remains controversial [15-18,47-49]. The reduced endogenous IgG sialylation in our IVIG-resistant subjects may have a direct effect on their response to treatment, or may simply be a marker for reduced ST6Gal-I activity. Thus, future research should focus on sialylation of other proteins that may be affecting treatment response.

In our study, sialic acid levels on the infused IVIG were low, and only one of the three assays showed a lower level of sialic acid on the IVIG received by IVIG-resistant subjects (Figure **3A-C**). However, in mice, administration of hyper-sialylated IVIG prevented arthritis at a ten-fold lower dose compared to unsialylated IVIG [12]. Based on our data, there does not appear to be a relationship between sialylation of IVIG and treatment response in our human KD patients. 

Gal and fucose modification of *N*-glycans on IgG are known to be important in the modulation of inflammation in murine models and human disease [10,40,43,44,50]. Levels of galactosylated IgG in KD were significantly higher in IVIG-resistant subjects compared to IVIG-responsive subjects at both the acute and one-year time points (Figure **3D**), which is the opposite of what was seen for sialic acid, as expected.. Total Gal levels were much higher than sialic acid levels, which suggests that only a minority of the terminal Gal molecules were sialylated. Galactosylation plays a role in complement activation by masking the terminal GlcNAc that binds to mannose-binding lectin (MBL), a first step in complement activation [10]. However, the role of complement in KD pathogenesis is uncertain so the significance of increased galactosylation in IVIG-resistant subjects it is unclear. The role of fucose in immune regulation is demonstrated by the 100-fold increase in affinity of the activating FcγRIIIA on NK cells and IIIb on neutrophils for all classes of human IgG following removal of the core fucose residue from the *N*-glycan in the IgG Fc region [43,51-53]. We found no significant differences in the levels of IgG fucosylation between KD subjects at the one-year time point and healthy control subjects and between acute KD subjects and febrile control subjects (Figure **3E**). Therefore, fucosylation of IgG does not appear to play a major role in KD pathogenesis or response to therapy.

The predominant sialic acid linkage in human IgG Fc glycans is α2-6 linkage, although Sia can also be found in a α2-3 linkage to the penultimate Gal on the Fc [13,22,42]. Using Western blotting, we detected only α2-6-linked, and not α2-3 linked Sias on the Fc glycan of infused IVIG and endogenous IgG from KD subjects. In addition, α2-6Sia was detected only from the Fc, and not the F(ab')_2_ portion of the IgG molecule (Figure **S1B**) . Our GC-MS measurement of α2-6Sias was consistent with the low α2-6Sia levels in IVIG-resistant subjects measured in the DMB and 2AB assays (Figure **3A-C**). Although the majority of the *N*-glycans have been detected on the Fc region of IgG from healthy human sera [19,54], 15-20% of human IgG molecules contain sialylated glycans on the F(ab')_2_ variable regions [55,56]. In addition, glycans in the F(ab')_2_ region are enriched for the bisecting GlcNAc compared to those in the Fc region (Figure **1B**) [57,58]. Using the MALDI-TOF MAS technique, we detected bisecting GlcNAc in very few IgG samples from KD and control subjects as well as other minor structures from the F(ab')_2_ region (e.g. high mannose structures). However, the signal intensities for this type of *N*-glycan were very low (Figure **S3D-F**). From these data, we conclude that the majority of the sialic acids in our IgG samples are derived from the *N*-glycan in the Fc region.

One possible explanation for the findings from our glycosylation analyses would be a genetic difference in sialylation enzymes between IVIG-responsive and -resistant subjects. Specifically, the lower α2-6Sia levels in endogenous IgG from IVIG-resistant subjects could be due to reduced transfer of Sias to the terminal Gal or to increased activity of enzymes that remove the terminal Sia from the *N*-glycans in IgG molecules (Figure **1B**). Expression levels of *ST6GAL1* were associated with IVIG treatment response in KD subjects (Figure **4** **, 5 and** Table **2**). The importance of ST6Gal-I in humoral immunity is well-established in both mice and humans [59-63]. Of the three known variants of human *ST6GAL1* transcripts, Transcript 1 is highly expressed in human hepatocytes, Transcript 2 is highly expressed in mature B cells, kidney, and spleen, and Transcript 3, appears to be ubiquitously expressed in various tissues (Figure **S2**) [33,34,64-66]. In mice, *ST6GAL1* transcribed from the hepatocyte-specific P1 promoter (Transcript 1) is critical for Fc sialylation of circulating IgG, even though Transcript 2 is abundantly expressed in mature B cells [20]. Although ST6Gal-I is usually found in the Golgi or plasma membrane, it can be proteolytically processed to a soluble form found in circulation [33,67]. ST6Gal-I also has anti-inflammatory effects by catalyzing sialylation of other molecules including receptors, lectins, and cytokines [24,25,29,30]. Studies in *ST6GAL1* P1 promoter knock-out mice have revealed an unexpected role of *ST6GAL1* Transcript 1 in regulating inflammation, with higher neutrophil response and G-CSF levels in knock-out mice following an intra-peritoneal inflammatory challenge [29]. G-CSF is a known biomarker for IVIG-resistance in KD, and elevated neutrophil counts are also associated with treatment resistance [68,69]. These observations could potentially be linked to the lower levels of ST6Gal-I that may lead to increased levels of G-CSF and increased numbers of circulating neutrophils in IVIG-resistant KD subjects. 

Microarray results were validated by qRT-PCR in the same subjects whose IgG sialylation had been measured. Amplification with primers specific for Transcript 2 showed the greatest difference in transcript levels. The levels of total ST6Gal-I and Transcript 2 from whole blood and B cell lines from IVIG-resistant subjects were consistently lower than those of responsive subjects (Figure **5A, C**, and **6A, C**). However, the expression levels of other ST6Gal-I transcripts varied to a lesser extent among subject groups (Figure **5B, D**, and Figure **5B**). There were no significant differences in whole blood transcript levels of the enzyme responsible for removing sialic acid, *NEU1*, between IVIG-responsive and -resistant subjects. The potential cellular sources of ST6Gal-I in human blood have not been rigorously studied.

Polymorphisms in human ST6Gal-I are associated with autoimmune and inflammatory conditions as well as atherosclerotic coronary artery disease in adults [63,70]. The persistently low levels of ST6Gal-I at one year in IVIG-resistant subjects suggest a genetic predisposition to a pro-inflammatory state influenced by variation in *ST6GAL1* expression. We are currently studying polymorphisms in *ST6GAL1* in these IVIG-resistant and –responsive subjects.

We recognize several strengths and limitations to our study. In this first analysis of IgG glycosylation patterns in children with KD and febrile and healthy children, we have discovered a potential biological mechanism to explain differences in response to IVIG treatment in KD subjects. We used three different analyses including DMB for total sialic acid, GC-MS for α2-6Sias, and 2AB for sialylated *N*-glycans. It was important to analyze these structures using a variety of experimental techniques with different assay principles to be confident of the results. All three methods showed low levels of sialic acid in endogenous IgG from IVIG-resistant KD subjects compared to IVIG-responsive subjects at both the acute (pre-treatment) and one-year time points. As blood volumes available from ill and anemic infants and young children were necessarily limited, we established B cell lines from these subjects as a surrogate for circulating B cells. Significant differences remained between IVIG-responsive and -resistant subjects. However, the expression of *ST6GAL1* may have been altered by EBV-mediated transformation [71]. We did not analyze IgG subclasses, whose distribution may vary with age and disease state, and glycosylation patterns may vary among subclasses [72]. The ability to generalize from our results is affected by the small sample size. Our findings should be viewed as hypothesis-generating and should be explored further in larger cohorts. While we found reproducible differences in sialylation of IgG and expression levels of *ST6GAL1* between the two KD study populations, further investigation will be required to uncover the genetic basis for these differences.

We found lower levels of α2-6Sia on endogenous IgG and lower ST6Gal-I levels in IVIG-resistant subjects at both the acute and one-year time points. Because IVIG-resistant KD patients have a higher rate of aneurysms, there is a clinical need to identify these subjects prior to IVIG administration and to target them with adjunctive treatment strategies [73,74]. Rapid assays for genotypes associated with reduced ST6Gal-I expression or for ST6Gal-I enzyme levels or activity could serve as clinically useful biomarkers for IVIG-resistance. 

## Supporting Information

Figure S1 (A) SDS-PAGE and Coomassie Blue stain of 3 μg IgG following papain digestion. Positive control: 3 μg purified human Fc fragment. (B) Western blots detecting Fc fragment, α2-6 linkage sialic acid and α2-3 linkage sialic on 3 μg IgG using anti-human Fc antibody, Biotinylated Sambucus nigra agglutinin (SNA) lectin, and Maackia amurensis lectin-II (MAL-II), respectively. 3 μg purified human Fc fragment, human albumin, and fetuin were used for negative and positive controls. IVIG: intravenous immunoglobulin, KD: Kawasaki disease.
(TIFF)

Figure S2 **Structure of ST6GAL1 mRNA.** ST6GAL1 has three different transcripts depending on promoter used in different cell types. Microarray clones were from exon VI (conserved region). Transcript levels of ST6GAL1 were analyzed with 4 different primer pairs. Our microarray clones were from exon VI (conserved region) of ST6GAL1 mRNA. Designed amplification with primers for the conserved region in exon VI, primer for Transcript 1 initiated from the P1 promoter (P1 promoter and exon I region, highly expressed in liver), primer for Transcript 2 (exon X and I region, highly expressed in spleen, kidney, and mature B cells), and primer for Transcript 3 (exons Y and Z, expressed in all cells).
(TIFF)

Figure S3 **Profiling N-glycans of infused IVIG and endogenous IgG from IVIG-responsive KD patient by 2AB labeling and MALDI-TOF assays.** (A) 2AB labeling profiles of given IVIG preparation (B) 2AB labeling profiles of endogenous IgG from IVIG-responsive at acute (pre-treatment) phase. (C) 2AB labeling profiles of endogenous IgG from IVIG-responsive at 1year phase after KD onset. (D) MALDI-TOF profiles of given IVIG preparation (E) MALDI-TOF profiles of endogenous IgG from IVIG-responsive at acute (pre-treatment) phase. (F) MALDI-TOF profiles of endogenous IgG from IVIG-responsive at 1year phase after KD onset. Blue square, GlcNAc; red triangle, fucose; green circle, mannose; yellow circle, galactose; purple diamond, α2-6 linkage sialic acid. All samples analyzed by glycobiology assays were verified by MALDI-TOF and 2AB assays. IVIG: intravenous immunoglobulin, KD: Kawasaki disease, 2AB: 2-aminobenzamide, MALDI-TOF: matrix-assisted laser desorption-ionization time-of-flight.
(TIFF)

Figure S4 **Correlation between levels of ST6GAL1 transcript and 2-6SA in IgG Coefficient of correlation was provided by the Speaman's correlations.** (A) Correlation between expression levels of total ST6GAL1 transcript (exon VI region) and 2-6SA levels in IgG, (B) Correlation between ST6GAL1 Transcript 2 (exon X and I region) and 2-6SA levels in IgG.
(TIFF)

Figure S5 **Soluble ST6Gal-I in culture supernatants from EBV-transformed B cell lines from 3 IVIG-responsive and 3 -resistant patients by ELISA assay.** KD: Kawasaki disease.
(TIFF)

Table S1 **Glycosylation assays for measurement of sialic acid in infused IVIG and endogenous IgG from IVIG-responsive and -resistant KD subjects.**
(DOC)

Table S2 **Characteristics of KD subjects for EBV- transformed B cell line experiments.**
(DOC)

Table S3 **Clinical characteristics and laboratory values at the acute time point for study subjects for ST6GAL1 experiments.**
(DOC)
