# Supplementary figures and images for: Treatment Response in Kawasaki Disease Is Associated with Sialylation Levels of Endogenous but Not Therapeutic Intravenous Immunoglobulin G
Source: PLoS One. 2013 Dec 6;8(12):e81448. doi: 10.1371/journal.pone.0081448 (PMC3855660; doi:10.1371/journal.pone.0081448)

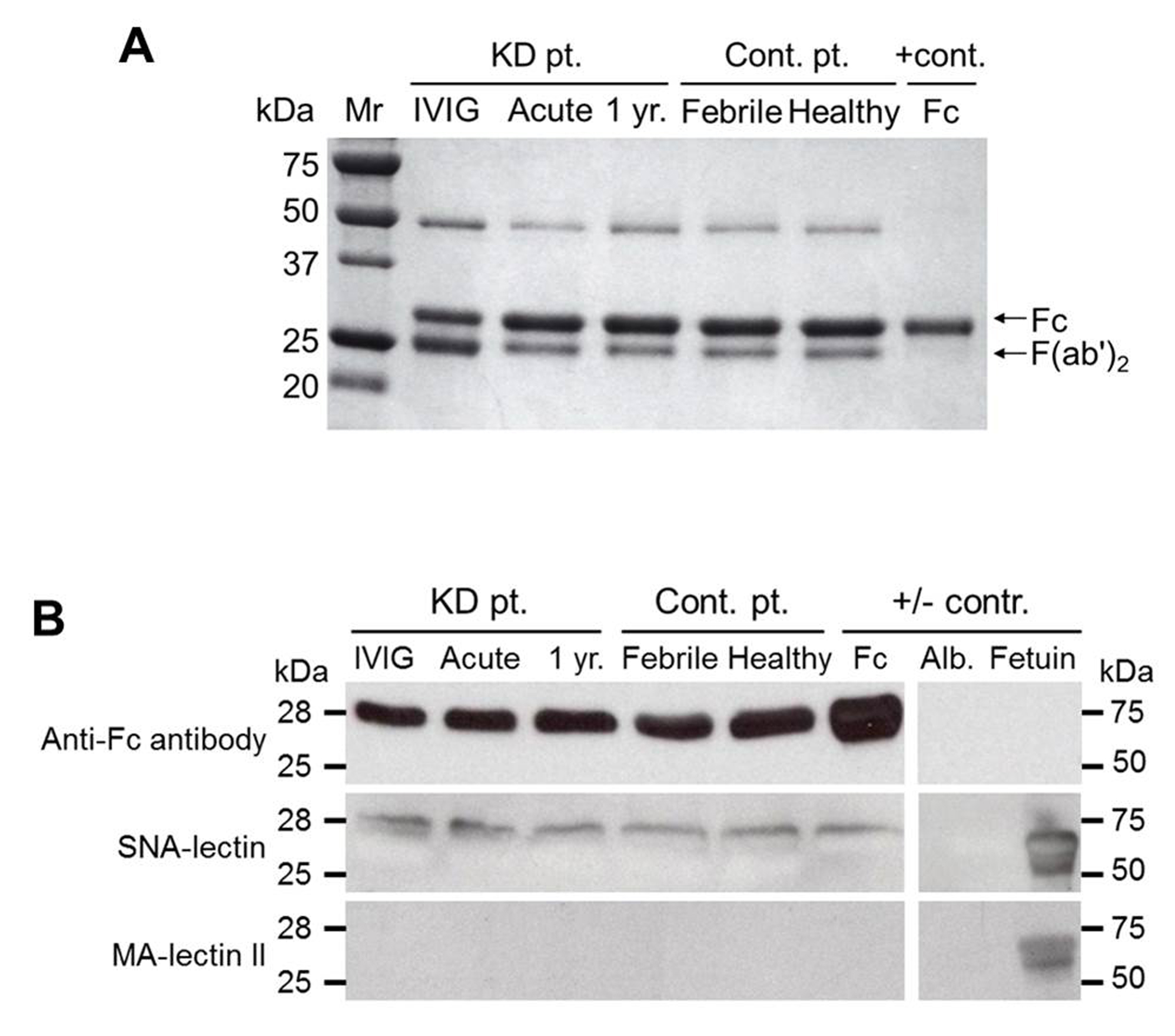

Supplement: Figure S1 — (A) SDS-PAGE and Coomassie Blue stain of 3 μg IgG following papain digestion. Positive control: 3 μg purified human Fc fragment. (B) Western blots detecting Fc fragment, α2-6 linkage sialic acid and α2-3 linkage sialic on 3 μg IgG using anti-human Fc antibody, Biotinylated Sambucus nigra agglutinin (SNA) lectin, and Maackia amurensis lectin-II (MAL-II), respectively. 3 μg purified human Fc fragment, human albumin, and fetuin were used for negative and positive controls. IVIG: intravenous immunoglobulin, KD: Kawasaki disease. (TIFF) [file pone.0081448.s001.tiff]

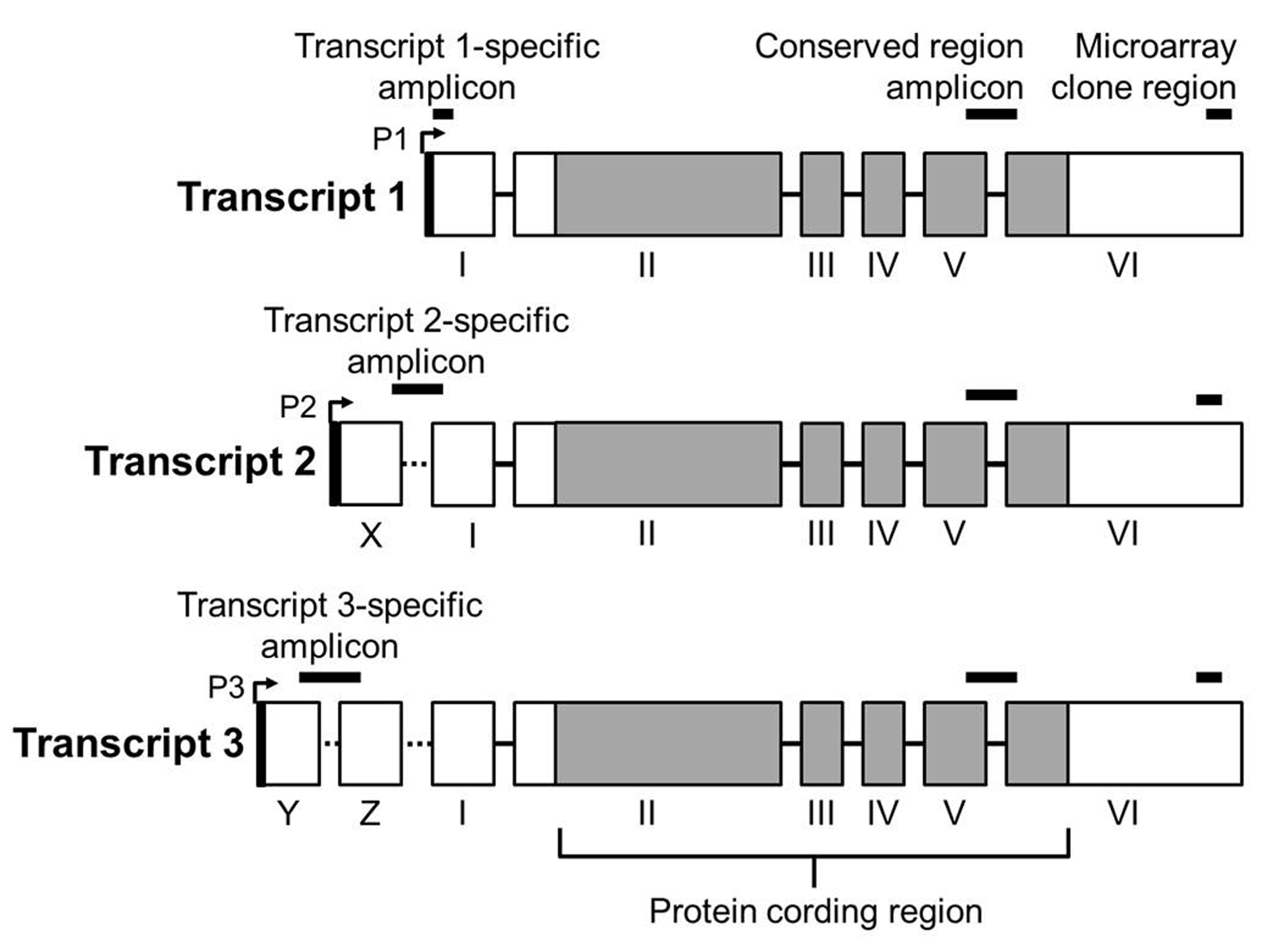

Supplement: Figure S2 — Structure of ST6GAL1 mRNA. ST6GAL1 has three different transcripts depending on promoter used in different cell types. Microarray clones were from exon VI (conserved region). Transcript levels of ST6GAL1 were analyzed with 4 different primer pairs. Our microarray clones were from exon VI (conserved region) of ST6GAL1 mRNA. Designed amplification with primers for the conserved region in exon VI, primer for Transcript 1 initiated from the P1 promoter (P1 promoter and exon I region, highly expressed in liver), primer for Transcript 2 (exon X and I region, highly expressed in spleen, kidney, and mature B cells), and primer for Transcript 3 (exons Y and Z, expressed in all cells). (TIFF) [file pone.0081448.s002.tiff]

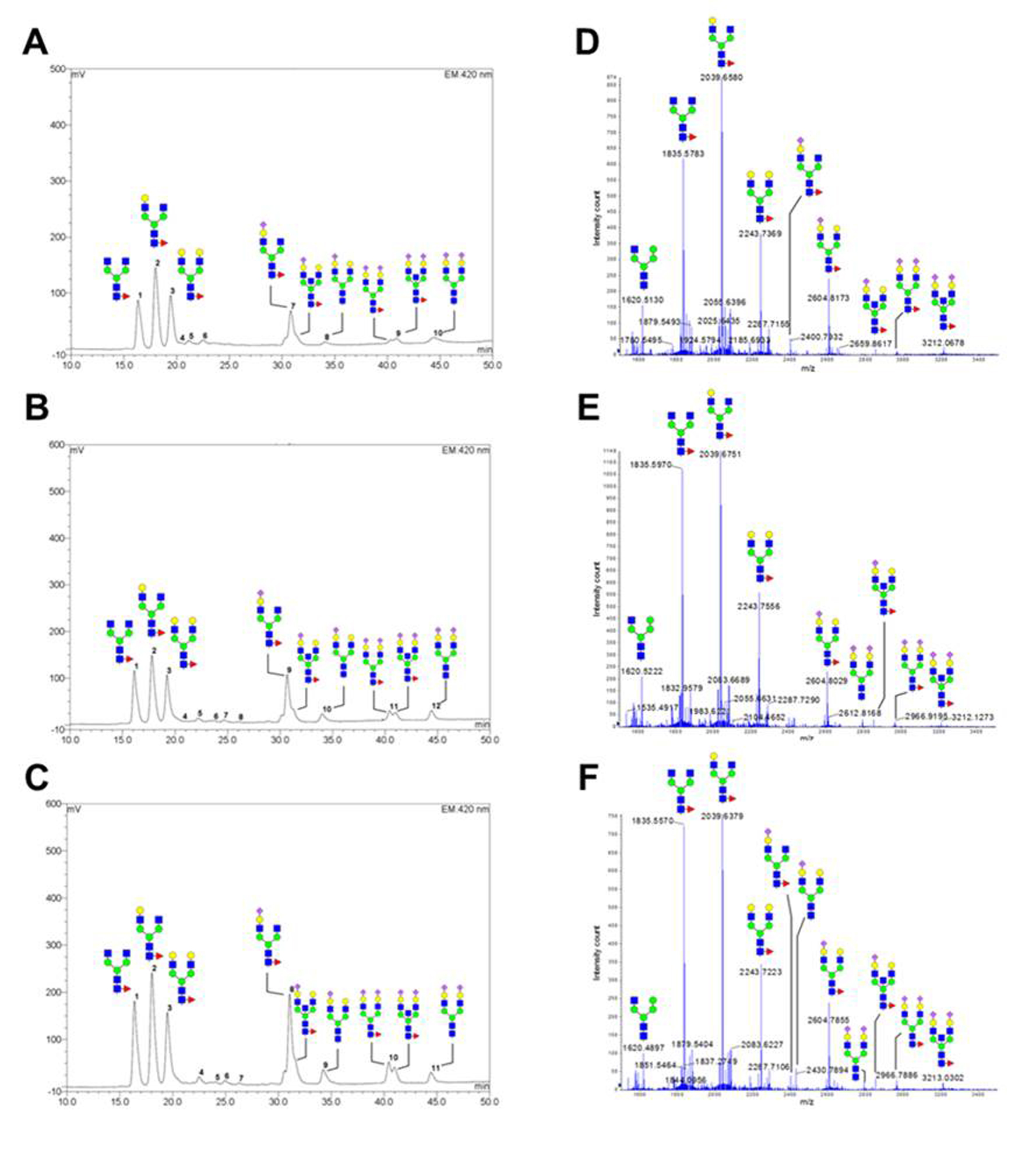

Supplement: Figure S3 — Profiling N-glycans of infused IVIG and endogenous IgG from IVIG-responsive KD patient by 2AB labeling and MALDI-TOF assays. (A) 2AB labeling profiles of given IVIG preparation (B) 2AB labeling profiles of endogenous IgG from IVIG-responsive at acute (pre-treatment) phase. (C) 2AB labeling profiles of endogenous IgG from IVIG-responsive at 1year phase after KD onset. (D) MALDI-TOF profiles of given IVIG preparation (E) MALDI-TOF profiles of endogenous IgG from IVIG-responsive at acute (pre-treatment) phase. (F) MALDI-TOF profiles of endogenous IgG from IVIG-responsive at 1year phase after KD onset. Blue square, GlcNAc; red triangle, fucose; green circle, mannose; yellow circle, galactose; purple diamond, α2-6 linkage sialic acid. All samples analyzed by glycobiology assays were verified by MALDI-TOF and 2AB assays. IVIG: intravenous immunoglobulin, KD: Kawasaki disease, 2AB: 2-aminobenzamide, MALDI-TOF: matrix-assisted laser desorption-ionization time-of-flight. (TIFF) [file pone.0081448.s003.tiff]

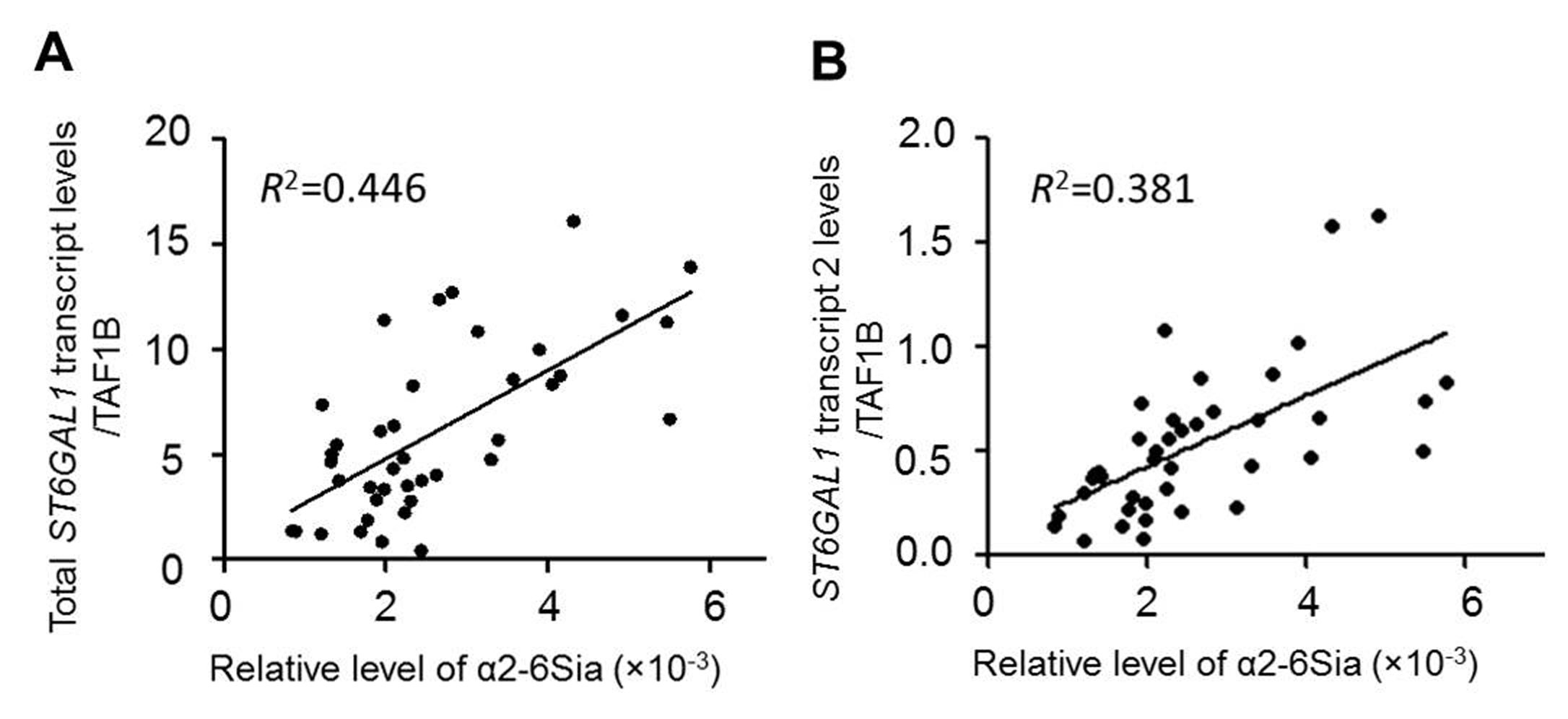

Supplement: Figure S4 — Correlation between levels of ST6GAL1 transcript and 2-6SA in IgG Coefficient of correlation was provided by the Speaman's correlations. (A) Correlation between expression levels of total ST6GAL1 transcript (exon VI region) and 2-6SA levels in IgG, (B) Correlation between ST6GAL1 Transcript 2 (exon X and I region) and 2-6SA levels in IgG. (TIFF) [file pone.0081448.s004.tiff]

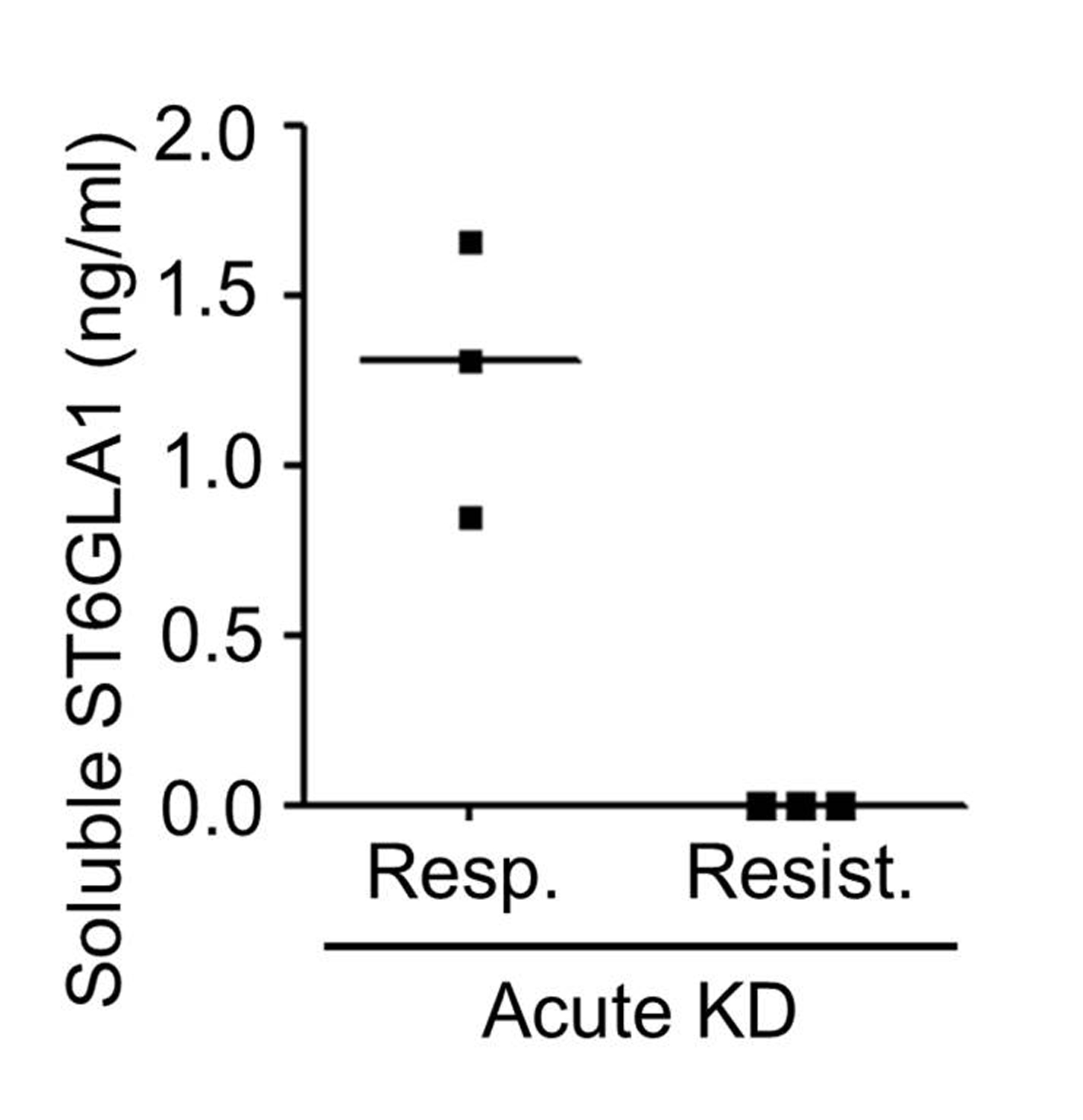

Supplement: Figure S5 — Soluble ST6Gal-I in culture supernatants from EBV-transformed B cell lines from 3 IVIG-responsive and 3 -resistant patients by ELISA assay. KD: Kawasaki disease. (TIFF) [file pone.0081448.s005.tiff]
