# Supplementary material for: Treatment Response in Kawasaki Disease Is Associated with Sialylation Levels of Endogenous but Not Therapeutic Intravenous Immunoglobulin G
Source: PLoS One. 2013 Dec 6;8(12):e81448. doi: 10.1371/journal.pone.0081448 (PMC3855660; doi:10.1371/journal.pone.0081448)
Supplement: Table S1 — Glycosylation assays for measurement of sialic acid in infused IVIG and endogenous IgG from IVIG-responsive and -resistant KD subjects. (DOC) [file pone.0081448.s006.doc]

**Supporting Tables**

**Table S1. Glycosylation assays for measurement of sialic acid in infused IVIG and endogenous IgG from IVIG-responsive and -resistant KD subjects**

|  | **IVIG preparation** | | | **Endogenous IgG from KD** | | | | | |
| --- | --- | --- | --- | --- | --- | --- | --- | --- | --- |
|  |  | | | **Acute** | | | **1 year** | | |
| **Assay** | **IVIG-responsive (n=10)** | **IVIG-resistant (n=10)** | **p** | **IVIG-responsive (n=10)** | **IVIG-resistant (n=10)** | **p** | **IVIG-responsive (n=10)** | **IVIG-resistant (n=10)** | **p** |
| DMB, ng/μg IgG (Total sialic acid) | 0.12 (0.12-0.13) | 0.12 (0.11-0.12) | NS | 0.22 (0.21-0.23) | 0.14 (0.11-0.17) | < 0.001 | 0.18 (0.15-0.19) | 0.14 (0.11-0.16) | 0.014 |
| 2AB, mV*min/μg IgG (Sialylated *N*-glycans) | 15.7 (14.8-18.4) | 12.8 (11.7-14.3) | 0.049 | 23.3 (21.9-26.8) | 18.5 (16.9-20.1) | 0.002 | 21.2 (18.1-24.4) | 17.5 (16.0-19.3) | 0.039 |
| GC-MS^A^, ×10^-3^ (α2-6 linked sialic acid) | 1.81 (0.67-2.29) | 6.34 (0.45-0.73) | NS | 3.97 (3.32-5.14) | 2.16 (1.96-2.30) | < 0.001 | 2.74 (2.22-3.46) | 1.32 (1.20-1.41) | < 0.001 |

Values are presented as median (IQR). p-values by Mann–Whitney U test. A. GC-MS values are shown as relative levels of α2-6 linked sialic acid/ 200 μg IgG/ 5 μg inositol. DMB: 1,2-diamino-4,5-methylenoxybenzene (DMB)-labeled sialic acid by HPLC, 2AB: 2-aminobenzamide, GC-MS: gas chromatography–mass spectrometry. KD: Kawasaki disease, IVIG: intravenous immunoglobulin; NS: Not significant.
