# Supplementary material for: Treatment Response in Kawasaki Disease Is Associated with Sialylation Levels of Endogenous but Not Therapeutic Intravenous Immunoglobulin G
Source: PLoS One. 2013 Dec 6;8(12):e81448. doi: 10.1371/journal.pone.0081448 (PMC3855660; doi:10.1371/journal.pone.0081448)
Supplement: Table S2 — Characteristics of KD subjects for EBV- transformed B cell line experiments. (DOC) [file pone.0081448.s007.doc]

**Supporting Tables**

**Table S2. Characteristics of KD subjects for EBV- transformed B cell line experiments**

|  | **IVIG-responsive (n=6)** | **IVIG-resistant (n=6)** | **p** |
| --- | --- | --- | --- |
| Age at diagnosis, years | 3.6 (2.7-4.1) | 3.7 (2.7-4.6) | NS |
| Male, n (%) | 4 (67) | 3 (50) | NS |
| *Illness day at sample collection, days | 6.5 (6.0-7.8) | 4.5 (3.3-5.8) | 0.021 |
| Coronary artery aneurysms, n (%) | 0 (0) | 1 (16.7) | NS |
| Ethnicity |  |  |  |
| Asian | 2 | 0 |  |
| Hispanic | 2 | 5 |  |
| More than race | 2 | 1 |  |

Values are presented as median (IQR). p-values by Mann–Whitney U test (continuous variables). and Fisher’s exact tests (categorical variables). ^*^Illness day 1: first calendar day of fever. KD: Kawasaki disease, IVIG: intravenous immunoglobulin NS: not significant
