# Supplementary material for: Treatment Response in Kawasaki Disease Is Associated with Sialylation Levels of Endogenous but Not Therapeutic Intravenous Immunoglobulin G
Source: PLoS One. 2013 Dec 6;8(12):e81448. doi: 10.1371/journal.pone.0081448 (PMC3855660; doi:10.1371/journal.pone.0081448)
Supplement: Table S3 — Clinical characteristics and laboratory values at the acute time point for study subjects for ST6GAL1 experiments. (DOC) [file pone.0081448.s008.doc]

**Supporting Tables**

**Table S3. Clinical characteristics and laboratory values at the acute time point for study subjects for ST6GAL1 experiments**

|  | **KD subjects** | | | **Control subjects** | |
| --- | --- | --- | --- | --- | --- |
|  | **IVIG-responsive (n=10)** | **IVIG-resistant (n=10)** | **p** | **Febrile control (n=10)** | **p^**^** |
| Age at diagnosis, years | 2.2 (2.0-3.6)^*^ | 1.9 (1.5-4.1) | NS | 1.3 (1.0-3.6) | NS |
| Male, n (%) | 4 (40) | 6 (60) | NS | 7 (70) | NS |
| ***Illness day at sample collection, days | 6 (5-7) | 4 (4-5) | NS | 4 (3-6) | NS |
| Coronary artery aneurysms, n (%) | 1 (10) | 2 (20) | NS | NA |  |
| Ethnicity, n |  |  |  |  |  |
| Asian | 0 | 0 |  | 1 |  |
| African-American | 1 | 1 |  | 2 |  |
| Caucasian | 4 | 2 |  | 2 |  |
| Hispanic | 5 | 4 |  | 3 |  |
| More than race | 4 | 3 |  | 2 |  |
| CRP, mg/dl | 7.5 (3.7-13.9) | 6.0 (4.8-16.0) | NS | 0.7 (0.5-5.6) | 0.010 |
| ESR, mm/h | 69 (41-79) | 50 (33-60) | NS | 31 (25-36) | 0.015 |
| WBC, ×10^3^/mm^3^ | 14.8 (11.9-16.7) | 11.7 (9.8-13.6) | NS | 8.1 (4.9-11.0) | 0.042 |
| Absolute neutrophil count | 11857 (8874-13653) | 8156 (6733-10226) | NS | 2656 (2002-5284) | 0.009 |
| Platelet count, ×10^3^/mm^3^ | 413 (348-457) | 328 (280-368) | NS | 277 (190-392) | NS |
| ALT, IU/l | 72 (55-134) | 87 (41-148) | NS | 21 (17-29) | 0.013 |
| GGT, IU/l | 66 (27-232) | 101 (34-165) | NS | 14 (12-17) | < 0.001 |

^*^Values are presented as median (IQR). p-values were calculated by Mann–Whitney U test for continuous variables and Fisher’s exact tests for categorical variables. ^**^The p-values were obtained by the comparison of KD patients with control patients.^***^Illness day 1: first calendar day of fever. All KD patients were complete KD. KD: Kawasaki disease, IVIG: Intra venous immunoglobulin G therapy CRP: C-reactive protein (Normal range: <0.5 mg/dl),, ESR: Erythrocyte sedimentation rate, WBC: White blood count, ALT: Alanine aminotransferase (Normal range: 10-25 IU/L), GGT: Gamma-glutamyltransferase(Normal range: 10-22 IU/L), NA: Not applicable, NS: Not Significant
